# Supplementary material for: Highly sensitive CE-ESI-MS analysis of N-glycans from complex biological samples
Source: Nat Commun. 2019 May 13;10:2137. doi: 10.1038/s41467-019-09910-7 (PMC6513864; doi:10.1038/s41467-019-09910-7)
Supplement: Supplementary file 1 — Supplementary Information [file 41467_2019_9910_MOESM1_ESM.pdf]

Supplementary Information

Highly sensitive CE-ESI-MS analysis of *N*-glycans from complex biological samples

Lageveen-Kammeijer and De Haan et al.

TABLE OF CONTENTS

Supplementary Figures 2

Supplementary Tables 25

Supplementrary References 29

SUPPLEMENTARY FIGURES

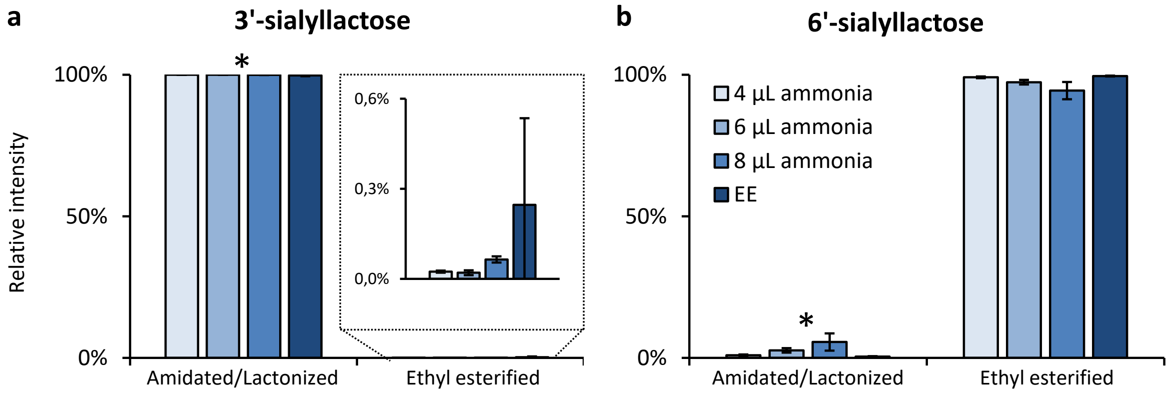

Supplementary Figure 1. Sialic acid linkage-specificity assessed by MALDI-TOF-MS. To robustly stabilize α2,3-linked sialic acids, an amidation step was added to the original ethyl esterification protocol[1] by adding a 28% ammonia solution to the reaction mixture after 1 h of incubation. Similar to the previously described double amidation (DA) reaction,[2] the ammonia hydrolyses the lactones on α2,3-linked sialic acids, after which they become and remain amidated. Different concentrations of ammonia were tested on 3'- and 6'-sialyllactose with 2-AB-label **(a** and **b)**, resulting in a maximum linkage specificity by adding 4 µL ammonia solution to the reaction mixture (0.02% non-specificity on 3'-sialyllactose and 0.9% non-specificity on 6'-sialyllactose). Error bars represent the standard deviation *(*$n = 4$*,* independent technical experiments*)*. * The α2,3-linked sialic acid was either amidated (addition of ammonia) or lactonized (ethyl esterification; EE). Source data are provided as a Source Data file.

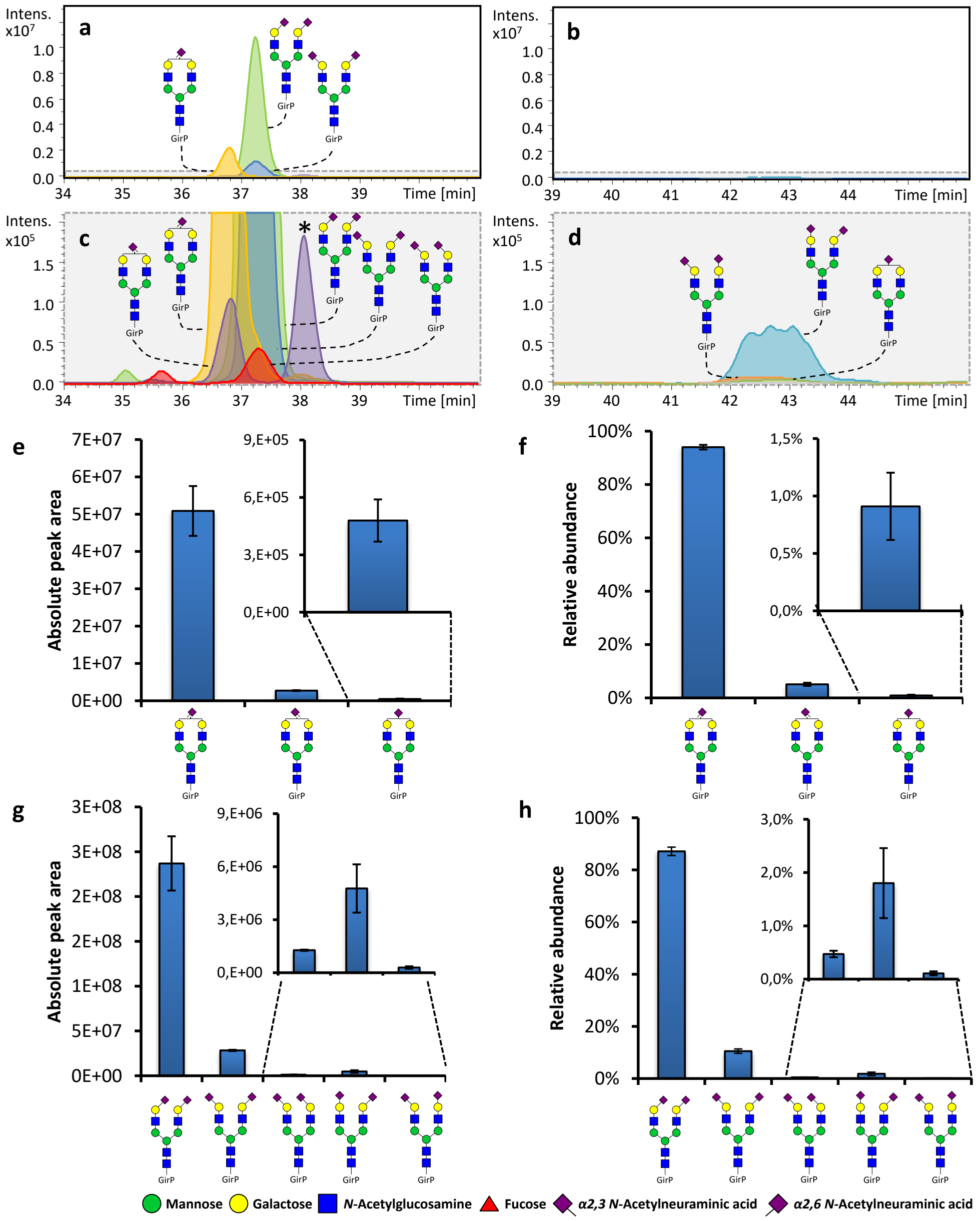

Supplementary Figure 2. **Derivatized and underivatized TPNG *N*-glycans.. (a-e)** Smoothed extracted ion electropherograms indicating the separation observed for diantennary mono- and disialylated TPNG *N*-glycans with various sialic acid linkage-combinations (with and without sialic acid derivatization), using a dynamically coated neutral BFS capillary. An overview of the *N*-glycan species at high intensity is provided **(a)** as well as an magnification, providing in-depth information for the low intensity *N*-glycans **(c)**. Underivatized *N*-glycans were observed at a high migration time and at a low intensity **(b & d)**. The absolute **(e-h)** and relative peak areas (**C**) reveal that the underivatized side products are of very low abundance (below 2.0%), **(e)** and **(f)** show monosialylated diantennary *N*-glycan species while **(g)** and **(h)** correspond to disialylated diantennary *N*-glycan species. CE-ESI-MS analysis was performed using DEN-gas. Blue square: *N*-acetylglucosamine, green circle: mannose, yellow circle: galactose, red triangle: fucose, right pointing pink diamond: α2,6-linked *N*-acetylneuraminic acid, left pointing pink diamond: α2,3-linked *N*-acetylneuraminic acid, up pointing pink diamond: non-derivatized *N*-acetylneuraminic acid of which the linkage could not be determined, GirP: Girard's reagent P label, attached to the *N*-glycans. * Indicates the presence of triply charged species of H6N5S$_{2,6}$3 of which the *m/z* values overlap with the ones of the doubly charged species of H5N4S$_{2,3}$1. Error bars represent the standard deviation *($n = 3$, independent technical experiments)*. Source data are provided as a Source Data file.

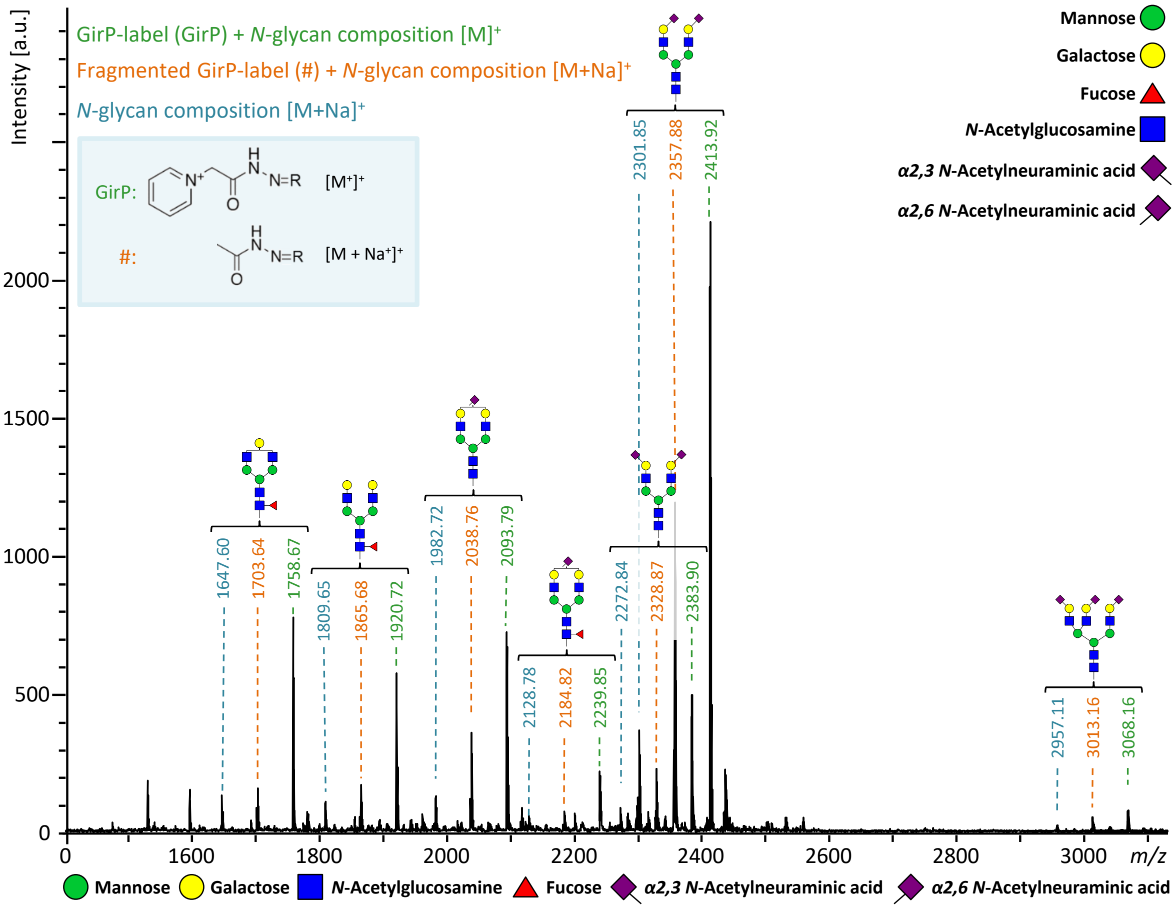

Supplementary Figure 3. MALDI-TOF-MS spectrum of dervitazed and labelled TPNG *N*-glycans. Annotated are the three signals of the seven glycoforms used to asses labelling efficiency (H4N4F1, H5N4F1, H5N4S$_{2,6}$1, H5N4F1S$_{2,6}$1, H5N4S$_{2,3}$1S$_{2,6}$1, H5N4S$_{2,6}$2, H6N5S$_{2,3}$1S$_{2,6}$2). Besides the GirP-labelled form of the molecules (GirP + *N*-glycan composition: [M$^+$]) and the unlabelled form of the molecules (*N*-glycan composition only: [M + Na]$^+$), also a fragmentation product of the labelled molecules was observed (# + *N*-glycan composition: [M + Na]$^+$). This fragmentation product was MALDI-specific and was added to the labelled fraction in the labelling-efficiency calculations. This fragmentation was not observed in the CE-ESI-MS analysis of the samples. A representative MALDI-TOF-MS spectrum is shown using the optimized conditions. Blue square: *N*-acetylglucosamine, green circle: mannose, yellow circle: galactose, red triangle: fucose, right pointing pink diamond: α2,6-linked *N*-acetylneuraminic acid, left pointing pink diamond: α2,3-linked *N*-acetylneuraminic acid, GirP: Girard's reagent P label, #: fragmented Girard's reagent P label.

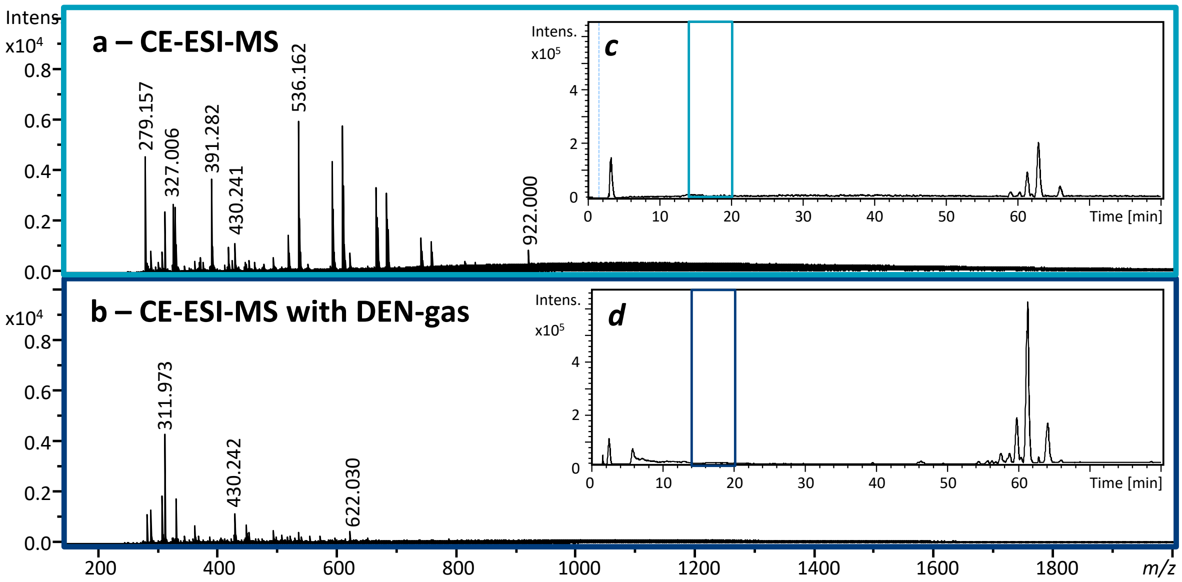

Supplementary Figure 4. **Observed background signal with and without DEN-gas using CE-ESI-MS. (a)** Summed MS spectrum obtained with a conventional CE-ESI-MS setup. **(b)** Summed MS spectrum obtained with CE-ESI-MS using DEN-gas. The base peak electropherograms corresponding to the analyses are **(c)** and **(d)**, the boxes indicate the time window over which the summed spectra were generated. Summed mass spectra were recorded between 14 and 20 min of the CE-ESI-MS analysis. Analysed samples were TPNG *N*-glycans after sialic acid derivatization (ethyl esterification and amidation) and reducing end labelling with GirP.

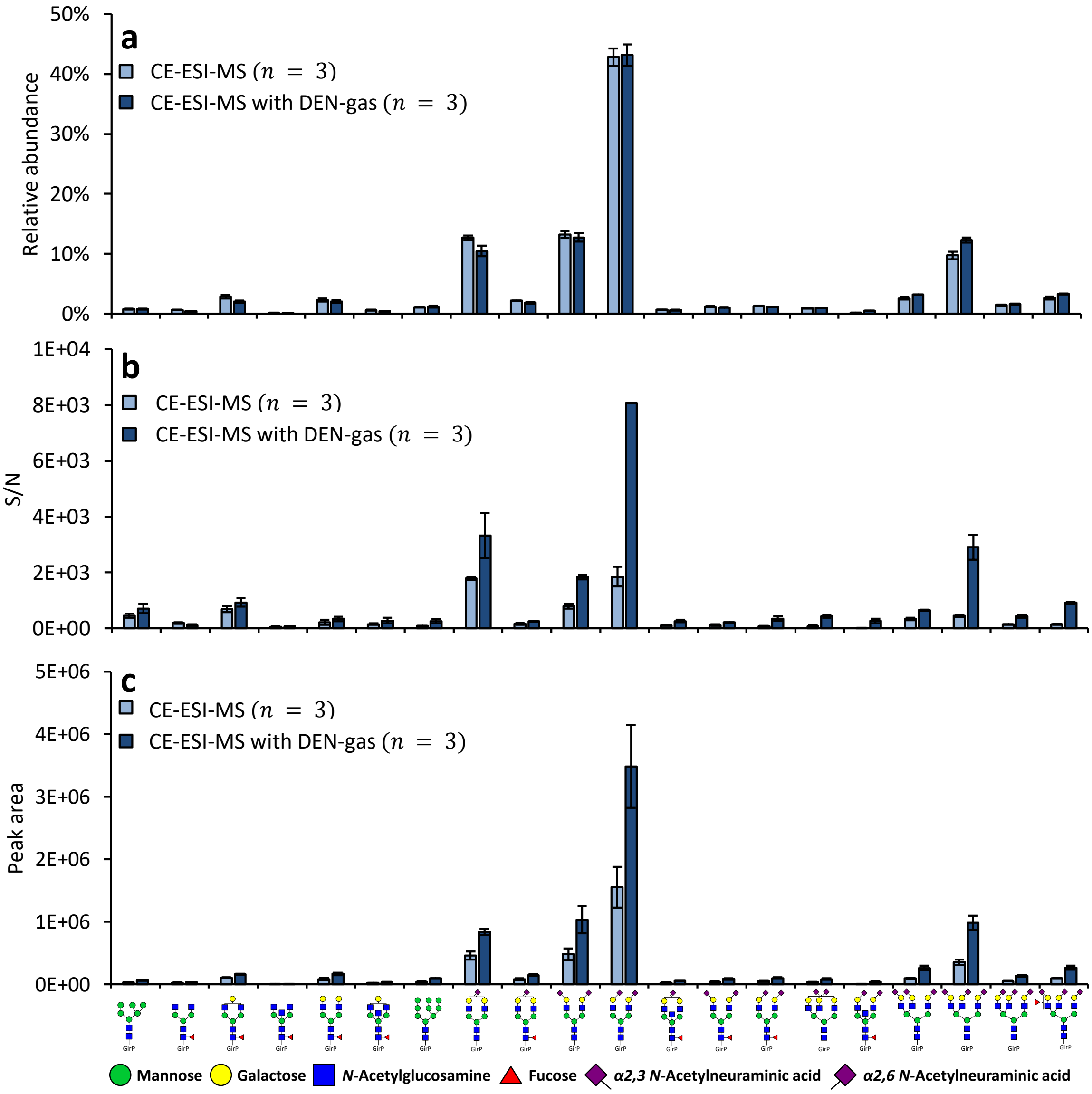

Supplementary Figure 5. Comparison of TPNG *N*-glycan analysis with CE-ESI-MS with or without DEN-gas. Relative abundances, signal-to-noise ratios (S/N) and absolute peak areas observed for the top 20 TPNG *N*-glycans after sialic acid derivatization (EEA) and reducing end labelling with GirP, between the CE-ESI-MS (light blue) and CE-ESI-MS with DEN-gas setup (dark blue). **(a)** Relative abundances, **(b)** S/N ratios and **(c)** absolute peak areas are shown. Integration was performed with LaCyTools.[3] Error bars represent the standard deviation ($n = 3$, independent technical experiments). Blue square: *N*-acetylglucosamine, green circle: mannose, yellow circle: galactose, red triangle: fucose, right pointing pink diamond: α2,6-linked *N*-acetylneuraminic acid, left pointing pink diamond: α2,3-linked *N*-acetylneuraminic acid, GirP: Girard's reagent P label, attached to the reducing end of the *N*-glycans. Source data are provided as a Source Data file.

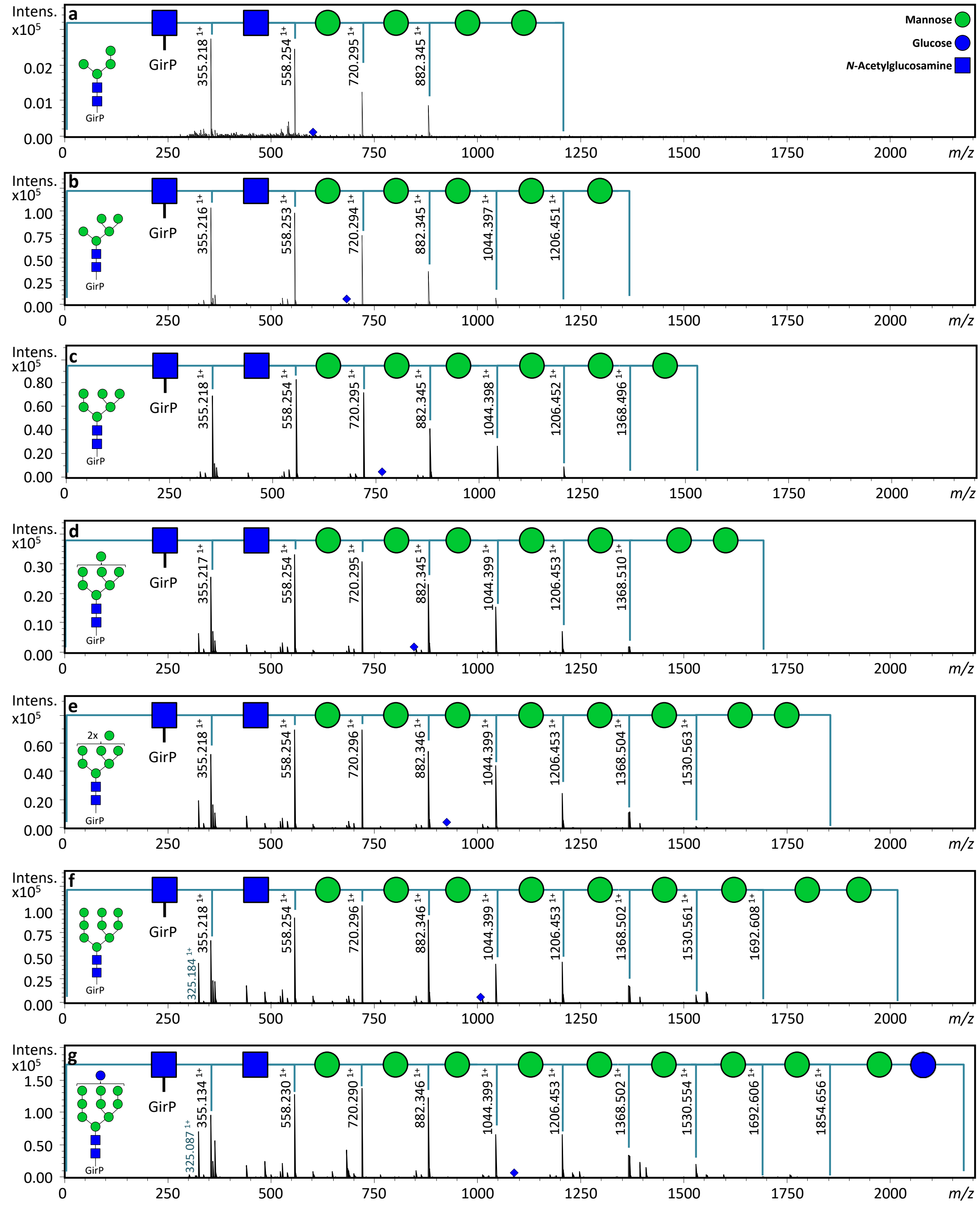

Supplementary Figure 6. CE-ESI-MS/MS fragmentation spectra of derivatized and labelled *N*-glycans. The following precursor ions were fragmented: **(a)** 603.739 $^{2+}$ (H4N2), **(b)** 684.758 $^{2+}$ (H5N2) **(c)** 765.782 $^{2+}$ (H6N2), **(d)** 846.808 $^{2+}$ (H7N2), **(e)** 927.833 $^{2+}$ (H8N2), **(f)** 1008.860 $^{2+}$ (H9N2) and **(g)** 1089.891 $^{2+}$ (H10N2). Blue diamond: precursor ion, blue square: *N*-acetylglucosamine, green circle: mannose, yellow circle: galactose, red triangle: fucose, right pointing pink diamond: α2,6-linked *N*-acetylneuraminic acid, left pointing pink diamond: α2,3-linked *N*-acetylneuraminic acid, GirP: Girard's reagent P label.

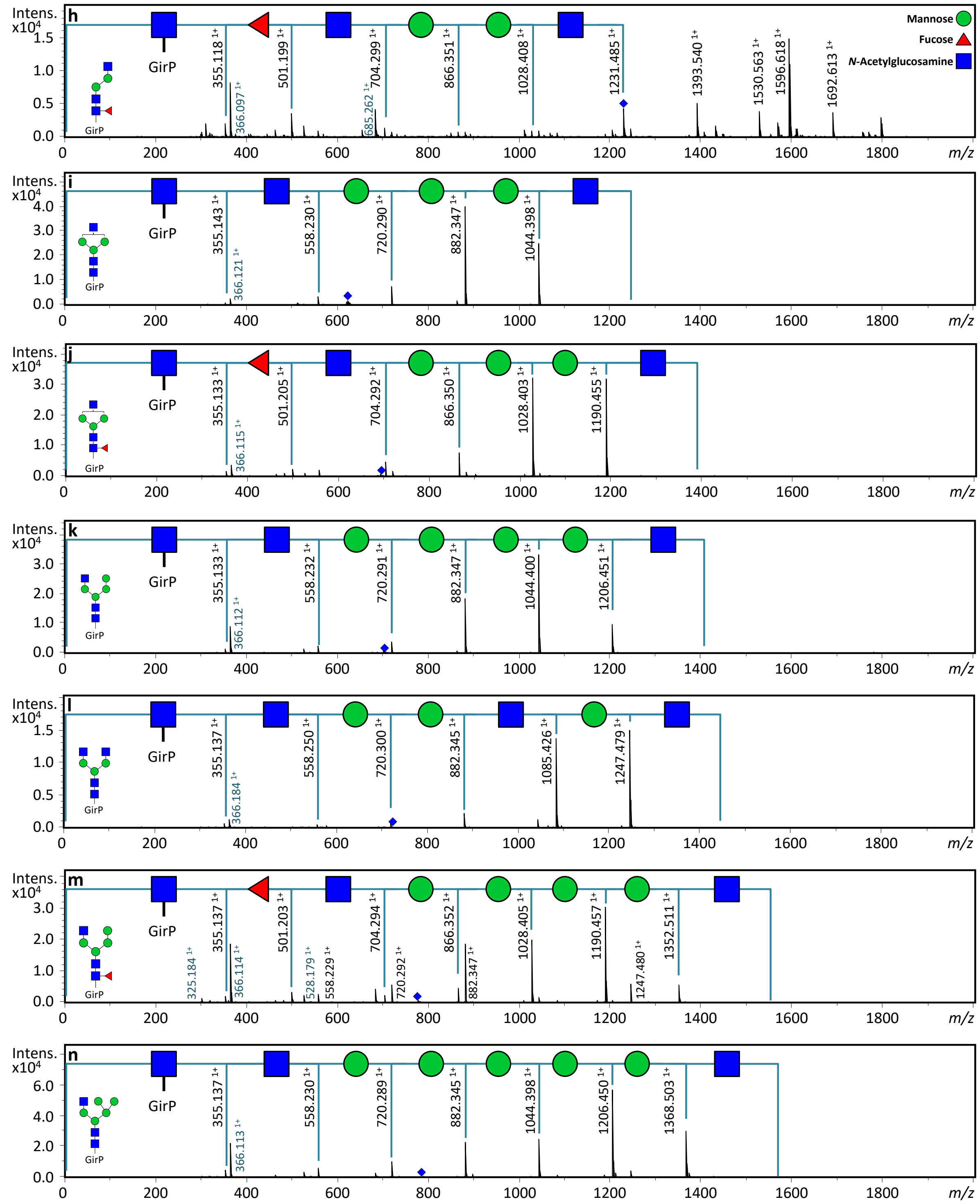

Supplementary Figure 6 (continued). CE-ESI-MS/MS fragmentation spectra of derivatized and labelled *N*-glycans. The following precursor ions were fragmented **(h)** 1231.485 $^{1+}$ (H2N3F1), **(i)** 624.236 $^{2+}$ (H3N3) **(j)** 697.267 $^{2+}$ (H3N3F1), **(k)** 705.267 $^{2+}$ (H4N3), **(l)** 725.782 $^{2+}$ (H3N4), **(m)** 778.296 $^{2+}$ (H4N3F1) and **(n)** 786.294 $^{2+}$ (H5N3). Blue diamond: precursor ion, blue square: *N*-acetylglucosamine, green circle: mannose, yellow circle: galactose, red triangle: fucose, right pointing pink diamond: α2,6-linked *N*-acetylneuraminic acid, left pointing pink diamond: α2,3-linked *N*-acetylneuraminic acid, GirP: Girard's reagent P label.

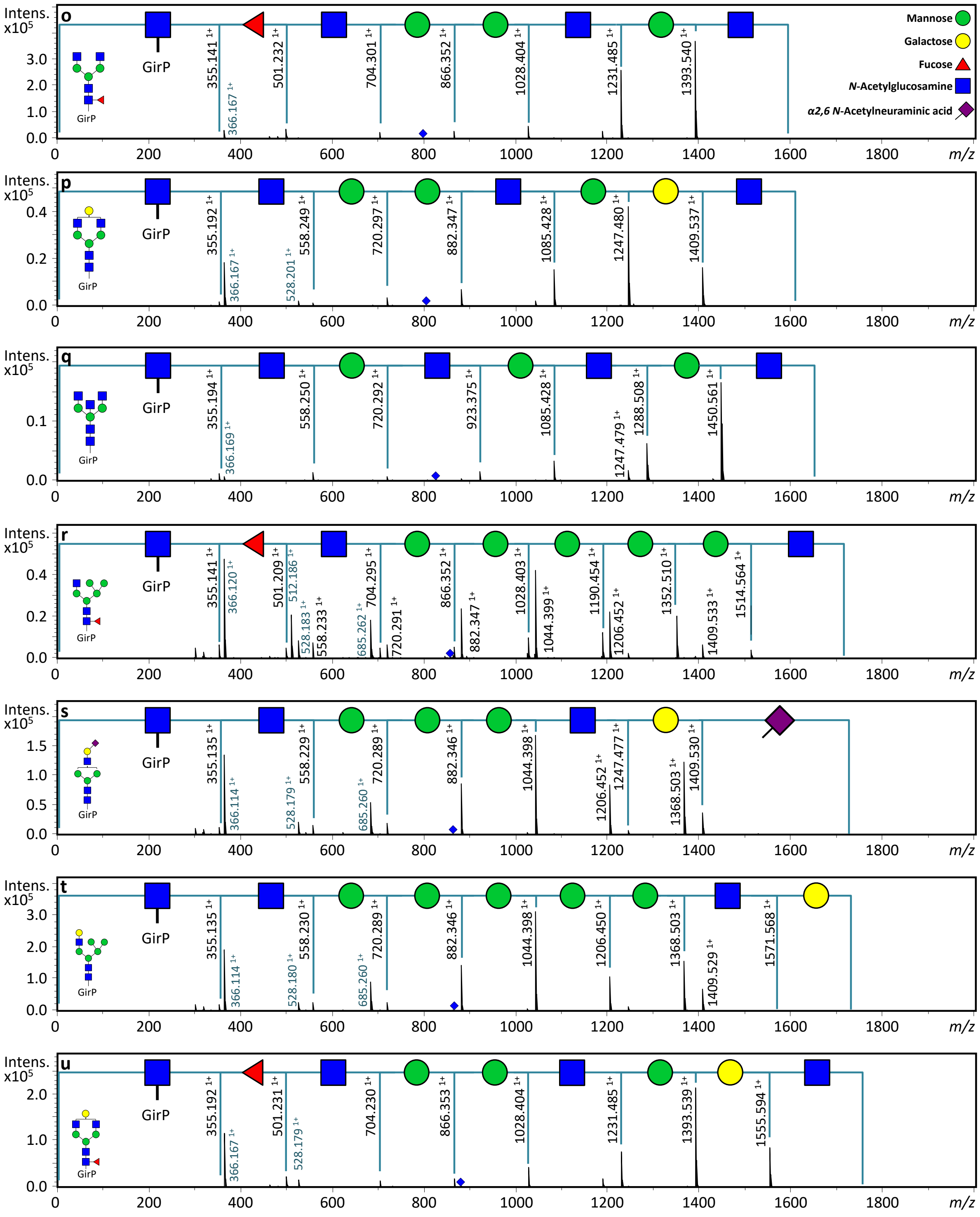

Supplementary Figure 6 (continued). CE-ESI-MS/MS fragmentation spectra of derivatized and labelled *N*-glycans. The following precursor ions were fragmented **(o)** 798.812 $^{1+}$ (H3N4F1), **(p)** 806.809 $^{2+}$ (H4N4) **(q)** 827.321 $^{2+}$ (H3N5), **(r)** 859.324 $^{2+}$ (H5N3F1), **(s)** 864.832 $^{2+}$ (H4N3S$_{2,6}$1), **(t)** 867.321 $^{2+}$ (H6N3) and **(u)** 879.837 $^{2+}$ (H4N4F1). Blue diamond: precursor ion, blue square: *N*-acetylglucosamine, green circle: mannose, yellow circle: galactose, red triangle: fucose, right pointing pink diamond: α2,6-linked *N*-acetylneuraminic acid, left pointing pink diamond: α2,3-linked *N*-acetylneuraminic acid, GirP: Girard's reagent P label.

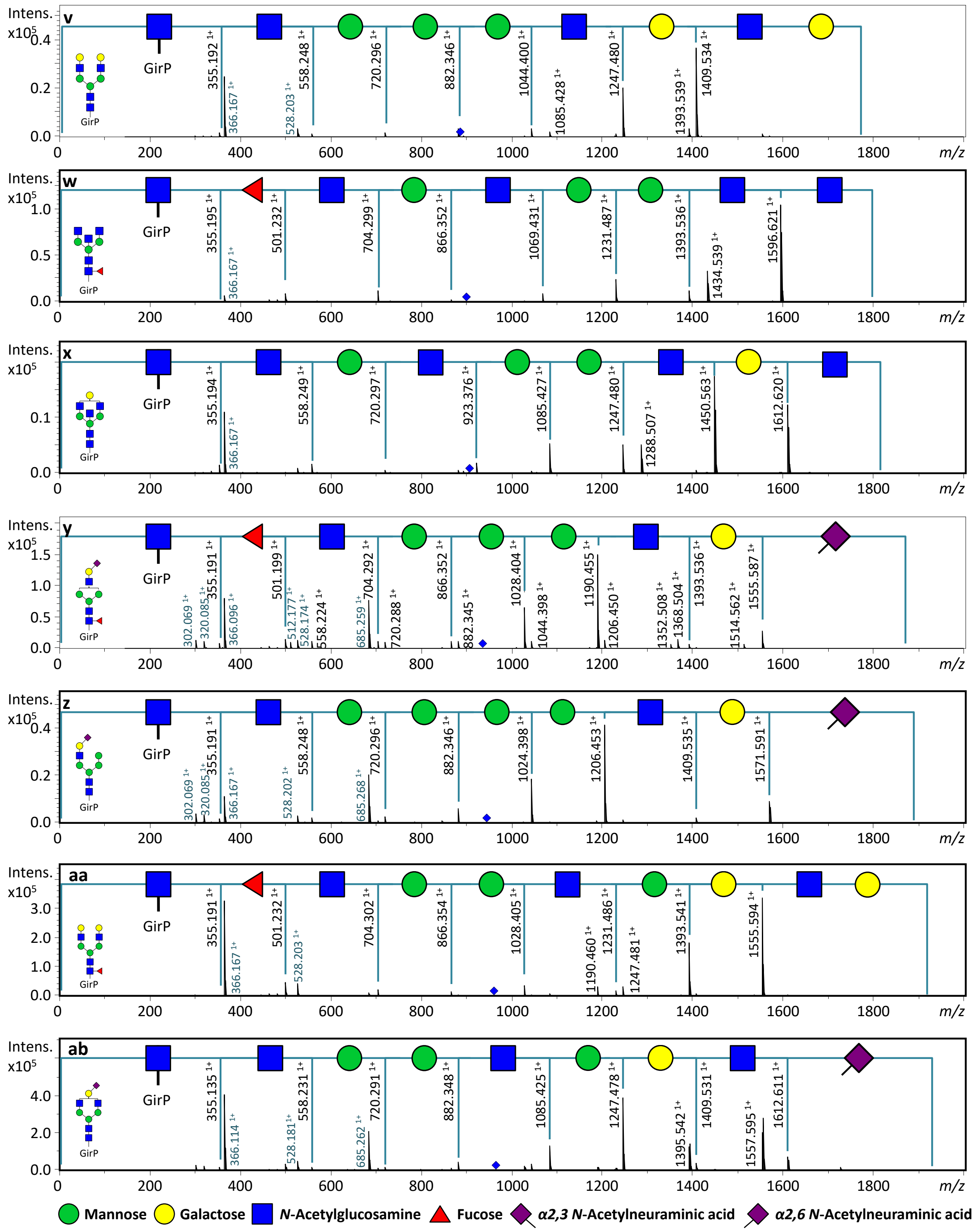

Supplementary Figure 6 (continued). CE-ESI-MS/MS fragmentation spectra of derivatized and labelled *N*-glycans. The following precursor ions were fragmented **(v)** 887.836 $^{2+}$ (H5N4), **(w)** 900.351 $^{2+}$ (H3N5F1) **(x)** 908.348 $^{2+}$ (H4N5), **(y)** 937.858 $^{2+}$ (H4N3F1S$_{2,6}$1), **(z)** 945.857 $^{2+}$ (H5N3S$_{2,6}$1), **(a)** 960.863 $^{2+}$ (H5N4F1) and **(ab)** 966.372 $^{2+}$ (H4N4S$_{2,6}$1). Blue diamond: precursor ion, blue square: *N*-acetylglucosamine, green circle: mannose, yellow circle: galactose, red triangle: fucose, right pointing pink diamond: α2,6-linked *N*-acetylneuraminic acid, left pointing pink diamond: α2,3-linked *N*-acetylneuraminic acid, GirP: Girard's reagent P label.

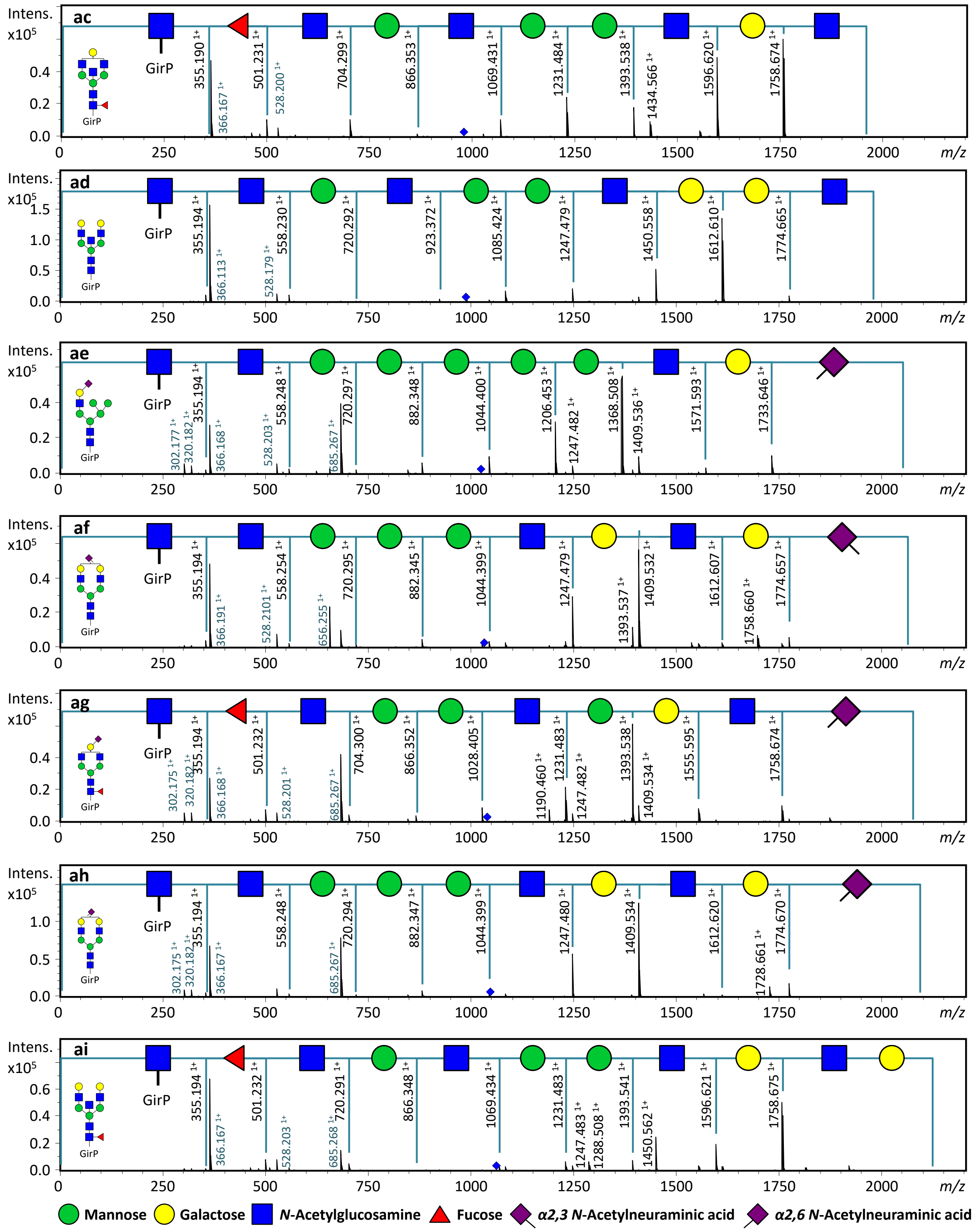

Supplementary Figure 6 (continued). CE-ESI-MS/MS fragmentation spectra of derivatized and labelled *N*-glycans. The following precursor ions were fragmented **(ac)** 981.375 $^{2+}$ (H4N5F1), **(ad)** 989.373 $^{2+}$ (H5N5) **(ae)** 1026.883 $^{2+}$ (H6N3S$_{2,6}$1), **(af)** 1032.890 $^{2+}$ (H5N4S$_{2,3}$1), **(ag)** 1039.399 $^{2+}$ (H4N4F1S$_{2,6}$1), **(ah)** 1047.396 $^{2+}$ (H5N4S$_{2,6}$1) and **(ai)** 1062.403 $^{2+}$ (H5N5F1). Blue diamond: precursor ion, blue square: *N*-acetylglucosamine, green circle: mannose, yellow circle: galactose, red triangle: fucose, right pointing pink diamond: α2,6-linked *N*-acetylneuraminic acid, left pointing pink diamond: α2,3-linked *N*-acetylneuraminic acid, GirP: Girard's reagent P label.

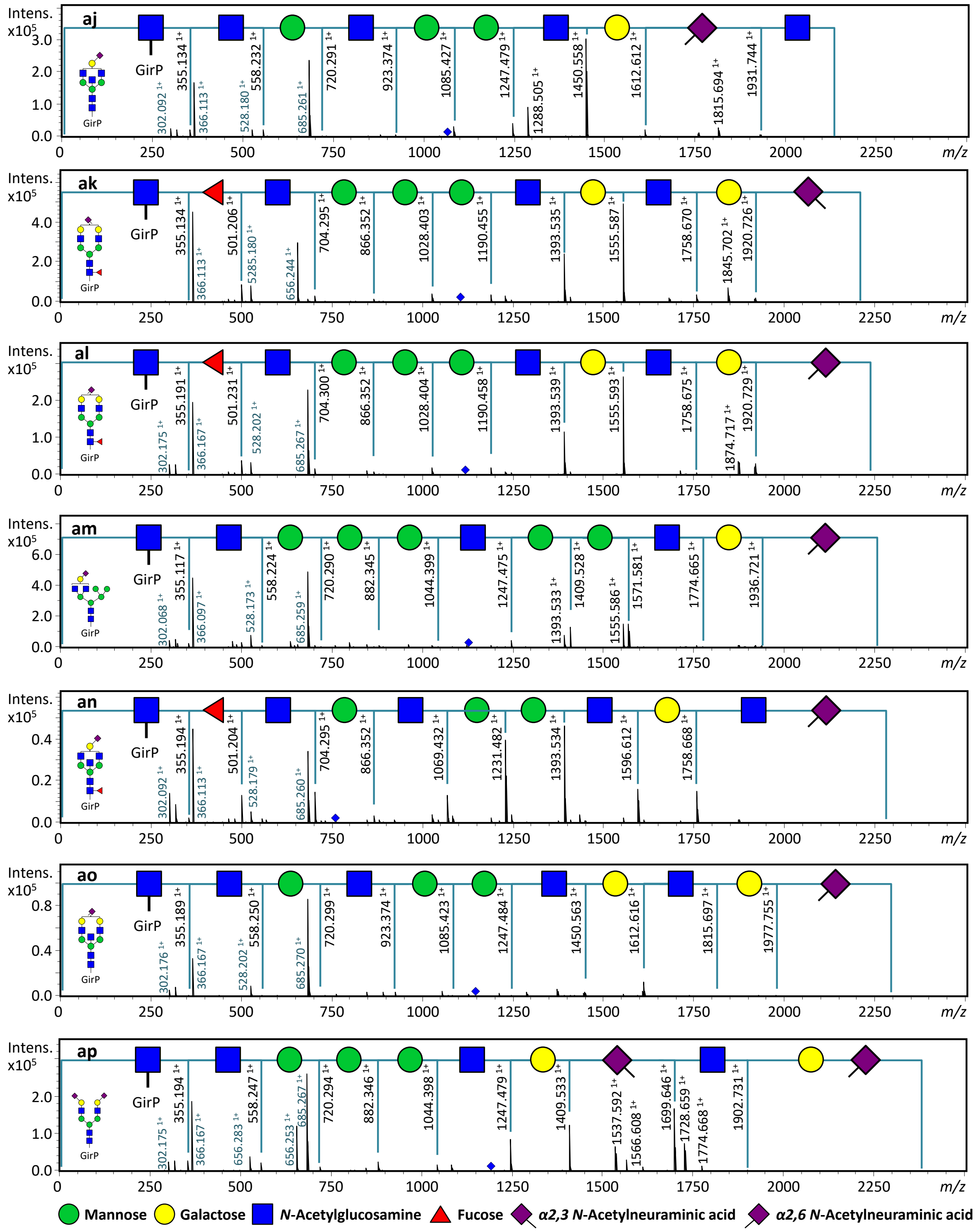

Supplementary Figure 6 (continued). CE-ESI-MS/MS fragmentation spectra of derivatized and labelled *N*-glycans. The following precursor ions were fragmented **(aj)** 1067.913 $^{2+}$ (H4N5S$_{2,6}$1), **(ak)** 624.236 $^{3+}$ (H5N4F1S$_{2,3}$1) **(al)** 697.267 $^{3+}$ (H5N4F1S$_{2,6}$1), **(am)** 705.267 $^{3+}$ (H6N4S$_{2,6}$1), **(an)** 725.782 $^{3+}$ (H4N5F1S$_{2,6}$1), **(ao)** 778.296 $^{3+}$ (H5N5S$_{2,6}$1) and **(ap)** 786.294 $^{3+}$ (H5N4S$_{2,3}$1S$_{2,6}$1). Blue diamond: precursor ion, blue square: *N*-acetylglucosamine, green circle: mannose, yellow circle: galactose, red triangle: fucose, right pointing pink diamond: α2,6-linked *N*-acetylneuraminic acid, left pointing pink diamond: α2,3-linked *N*-acetylneuraminic acid, GirP: Girard's reagent P label.

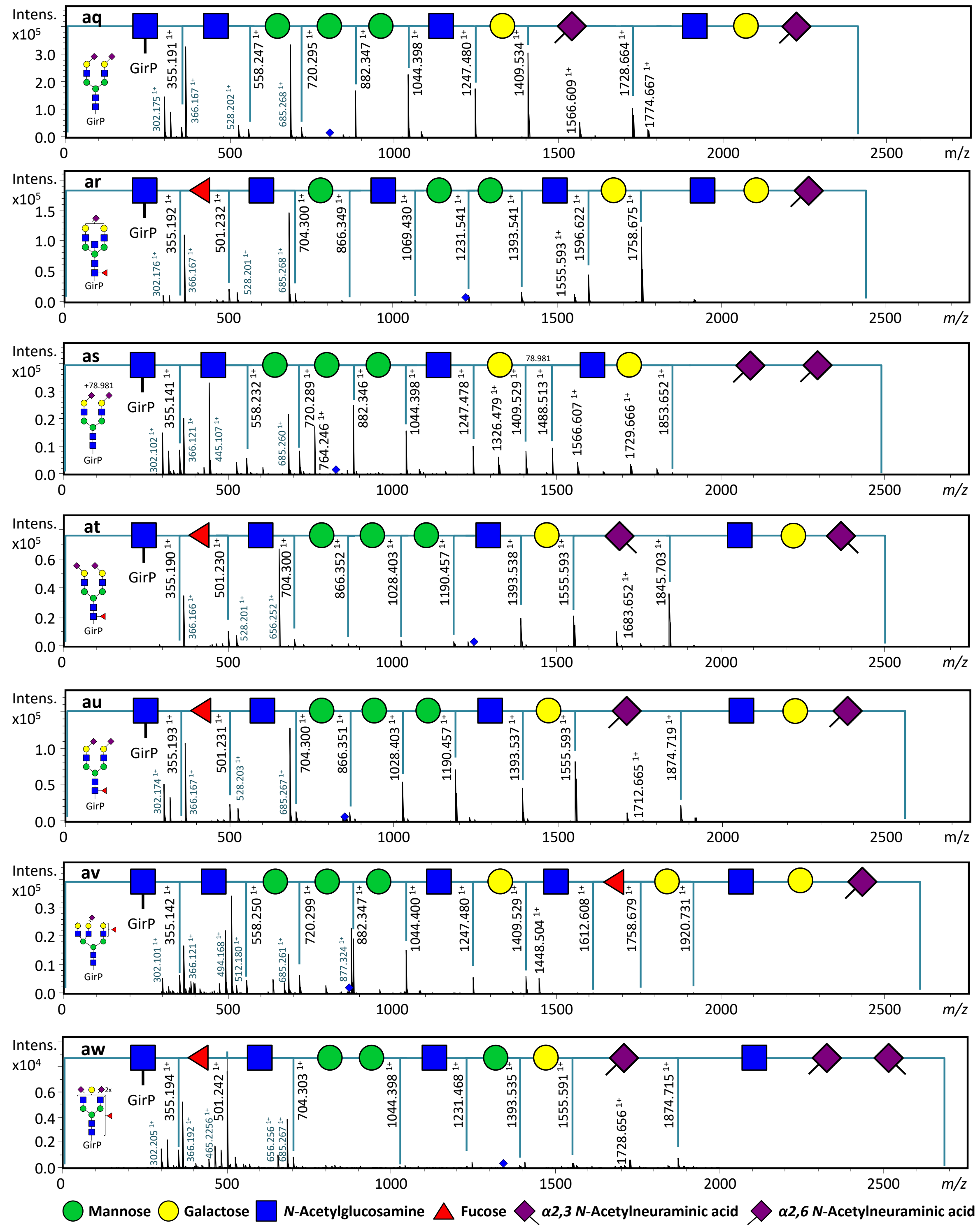

Supplementary Figure 6 (continued). CE-ESI-MS/MS fragmentation spectra of derivatized and labelled *N*-glycans. The following precursor ions were fragmented **(aq)** 804.978 $^{3+}$ (H5N4$S_{2,6}$2), **(ar)** 1221.967 $^{2+}$ (H5N5F1$S_{2,6}$1) **(as)** 831.304 $^{3+}$ (H5N4$S_{2,6}$2 [Adduct]), **(at)** 1250.975 $^{2+}$ (H5N4F1$S_{2,3}$)92), **(au)** 853.663 $^{3+}$ (H5N4F1$S_{2,6}$2), **(av)** 869.330 $^{3+}$ (H6N5F1$S_{2,6}$1) and **(aw)** 1344.010 $^{2+}$ (H4N4$S_{2,3}$1$S_{2,6}$2). Blue diamond: precursor ion, blue square: *N*-acetylglucosamine, green circle: mannose, yellow circle: galactose, red triangle: fucose, right pointing pink diamond: α2,6-linked *N*-acetylneuraminic acid, left pointing pink diamond: α2,3-linked *N*-acetylneuraminic acid, GirP: Girard's reagent P label.

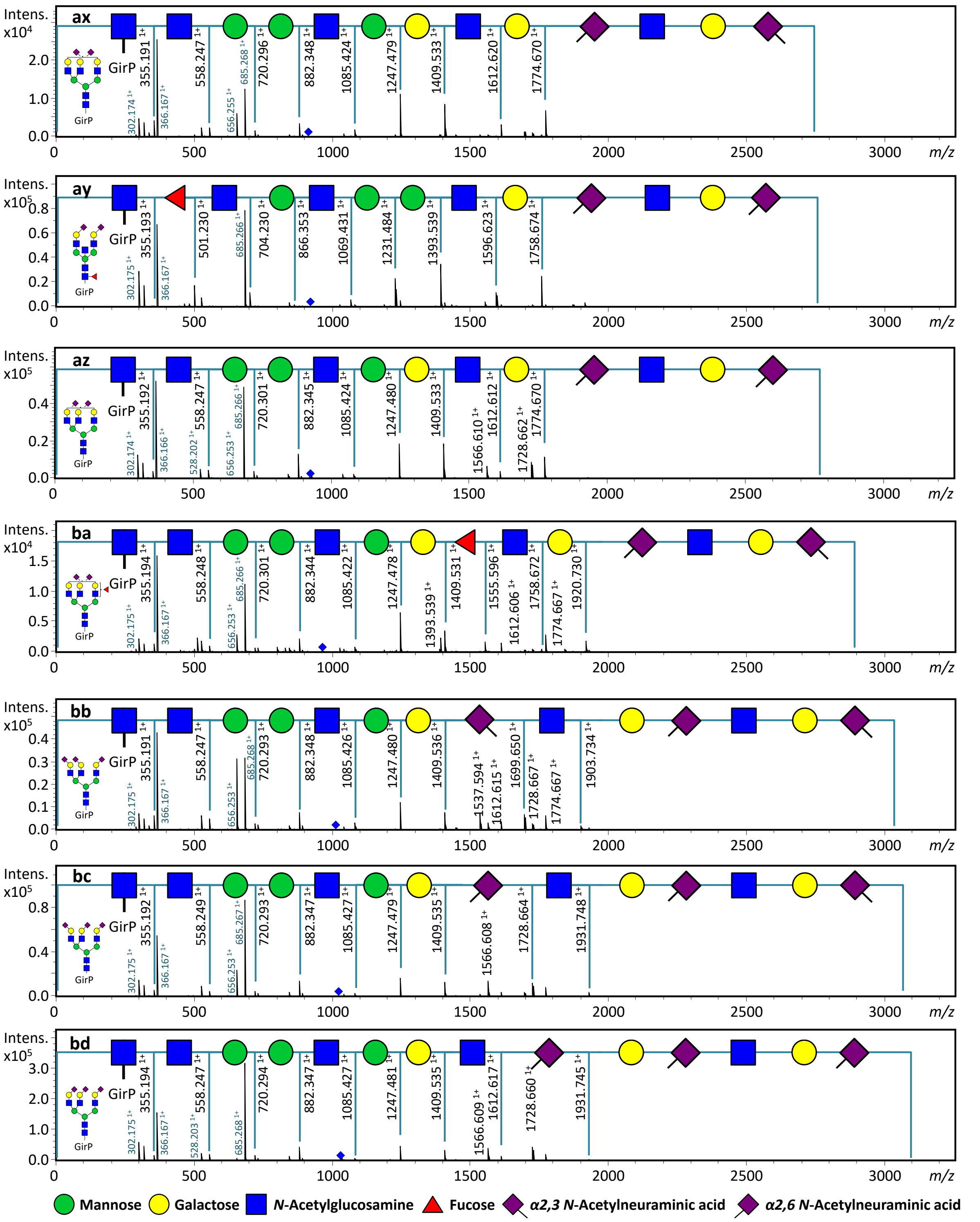

Supplementary Figure 6 (continued). CE-ESI-MS/MS fragmentation spectra of derivatized and labelled *N*-glycans. The following precursor ions were fragmented **(ax)** 917.0111 $^{3+}$ (H6N5 $S_{2,3}1S_{2,6}1$), **(ay)** 921.354 $^{3+}$ (H5N5F1$S_{2,6}2$) **(az)** 927.017 $^{3+}$ (H6N5$S_{2,6}2$), **(ba)** 966.033 $^{3+}$ (H6N5F1$S_{2,3}1S_{2,6}1$), **(bb)** 1014.049 $^{3+}$ (H6N5$S_{2,3}2S_{2,6}1$), **(bc)** 1023.721 $^{3+}$ (H6N5 $S_{2,3}1S_{2,6}2$) and **(bd)** 1033.059 $^{3+}$ (H6N5$S_{2,6}3$). Blue diamond: precursor ion, blue square: *N*-acetylglucosamine, green circle: mannose, yellow circle: galactose, red triangle: fucose, right pointing pink diamond: α2,6-linked *N*-acetylneuraminic acid, left pointing pink diamond: α2,3-linked *N*-acetylneuraminic acid, GirP: Girard's reagent P label.

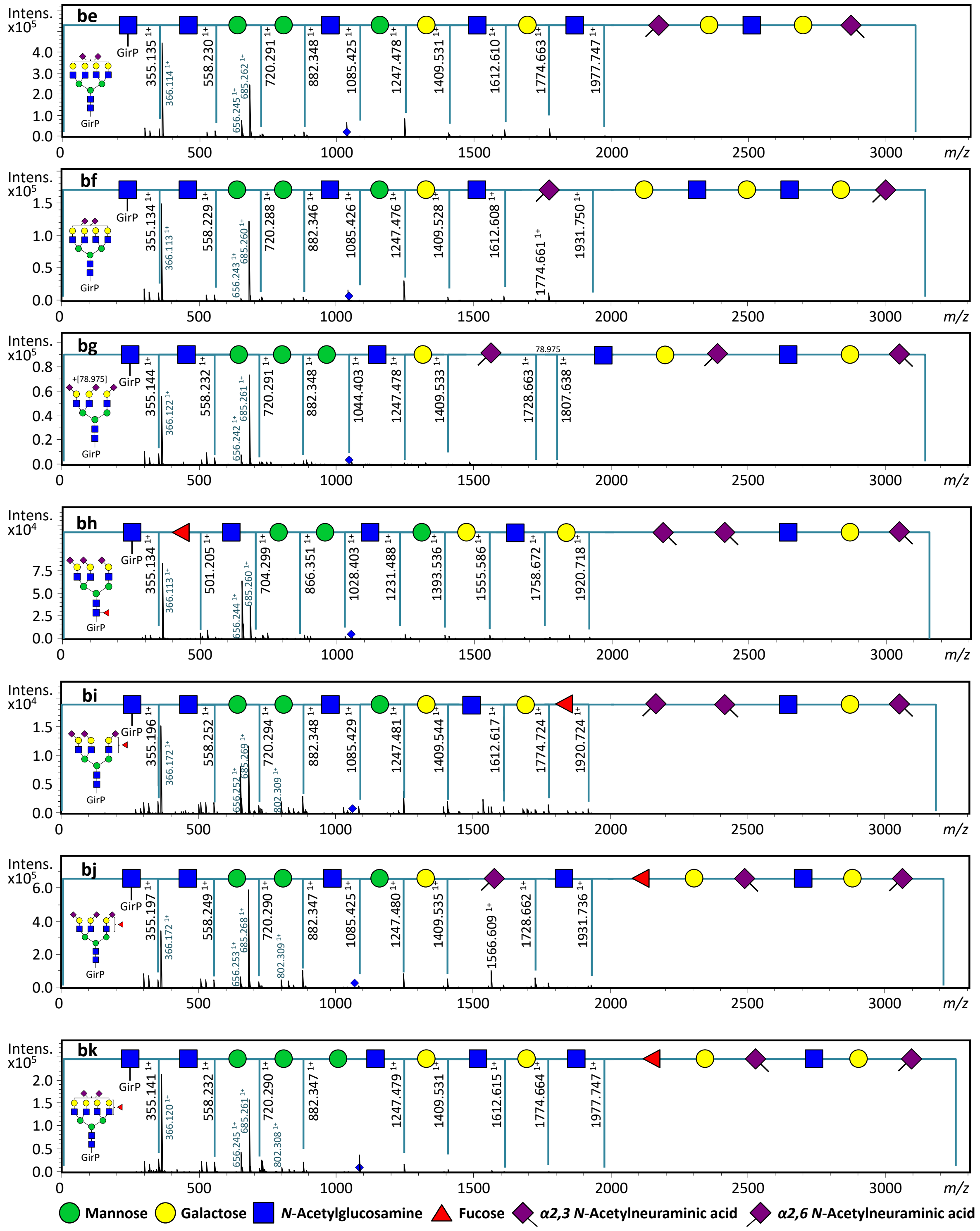

Supplementary Figure 6 (continued). CE-ESI-MS/MS fragmentation spectra of derivatized and labelled *N*-glycans. The following precursor ions were fragmented **(be)** 1039.062 $^{3+}$ ($H7N6S_{2,3}1S_{2,6}1$), **(bf)** 1048.732 $^{3+}$ ($H7N6S_{2,6}2$) **(bg)** 1050.052 $^{3+}$ ($H6N5S_{2,3}1S_{2,6}2^{[ADDUCT]}$), **(bh)** 1052.733 $^{3+}$ ($H6N5F1S_{2,3}3$), **(bi)** 1062.405 $^{3+}$ ($H6N5F1S_{2,3}2S_{2,6}1$), **(bj)** 1072.076 $^{3+}$ ($H6N5F1S_{2,3}1S_{2,6}2$) and **(bk)** 1087.411 $^{3+}$ ($H7N6F1S_{2,3}1S_{2,6}1$). Blue diamond: precursor ion, blue square: *N*-acetylglucosamine, green circle: mannose, yellow circle: galactose, red triangle: fucose, right pointing pink diamond: α2,6-linked *N*-acetylneuraminic acid, left pointing pink diamond: α2,3-linked *N*-acetylneuraminic acid, GirP: Girard's reagent P label.

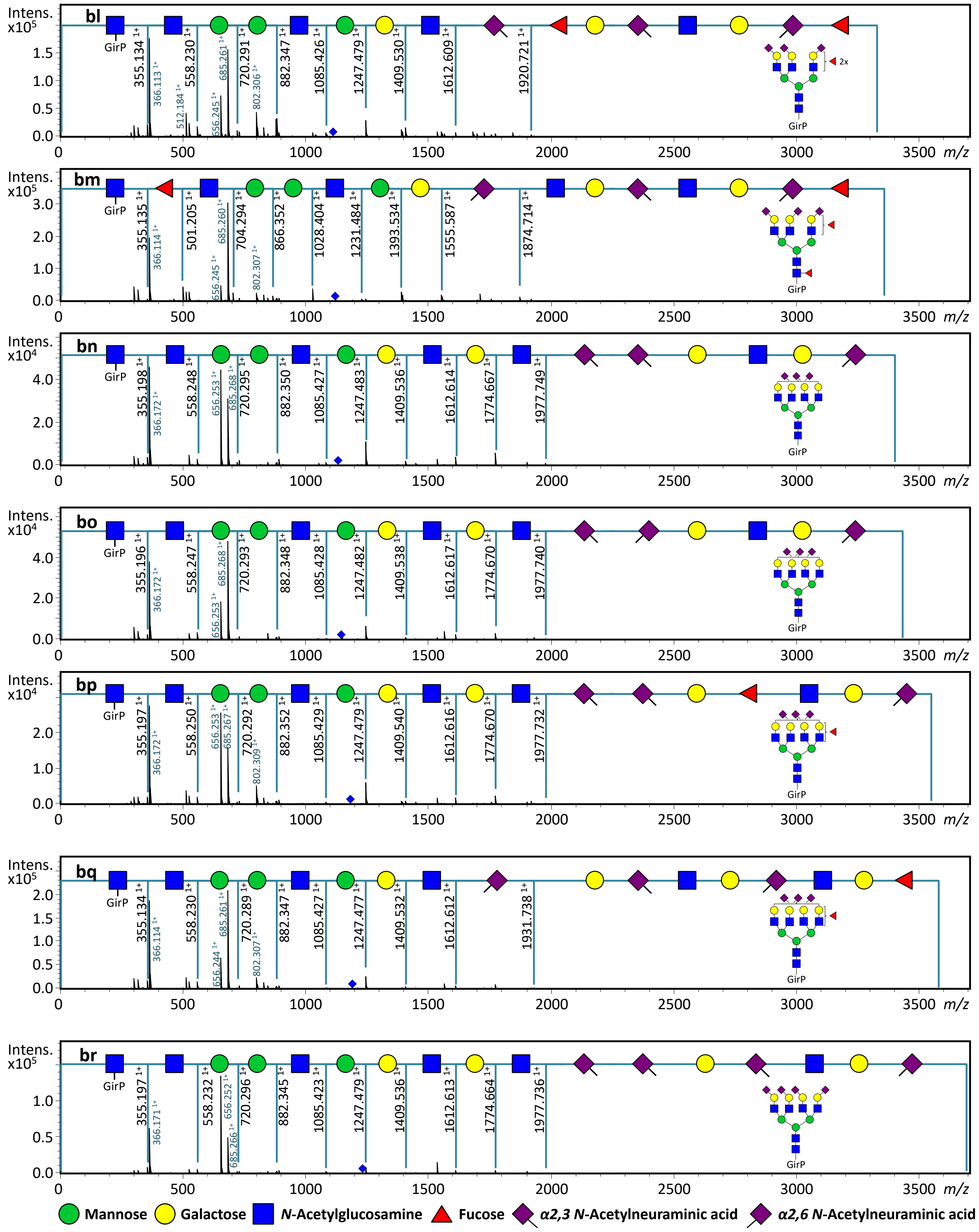

Supplementary Figure 6 (continued). CE-ESI-MS/MS fragmentation spectra of derivatized and labelled *N*-glycans from TPNG. The following precursor ions were fragmented **(bl)** 1111.424^{3+} ($H6N5F2S_{2,3}2S_{2,6}1$), **(bm)** 1121.096^{3+} ($H6N5F1S_{2,3}1S_{2,6}2$) **(bn)** 1135.428^{3+} ($H7N6S_{2,3}12S_{2,6}1$), **(bo)** 1145.100^{3+} ($H7N6S_{2,3}1S_{2,6}2$), **(bp)** 1184.115^{3+} ($H7N6F1S_{2,3}2S_{2,6}1$), **(bq)** 1194.121^{3+} ($H7N6F1S_{2,3}1S_{2,6}2$) and **(br)** 1232.130^{3+} ($H7N6S_{2,3}3S_{2,6}1$). Blue diamond: precursor ion, blue square: *N*-acetylglucosamine, green circle: mannose, yellow circle: galactose, red triangle: fucose, right pointing pink diamond: α2,6-linked *N*-acetylneuraminic acid, left pointing pink diamond: α2,3-linked *N*-acetylneuraminic acid, GirP: Girard's reagent P label.

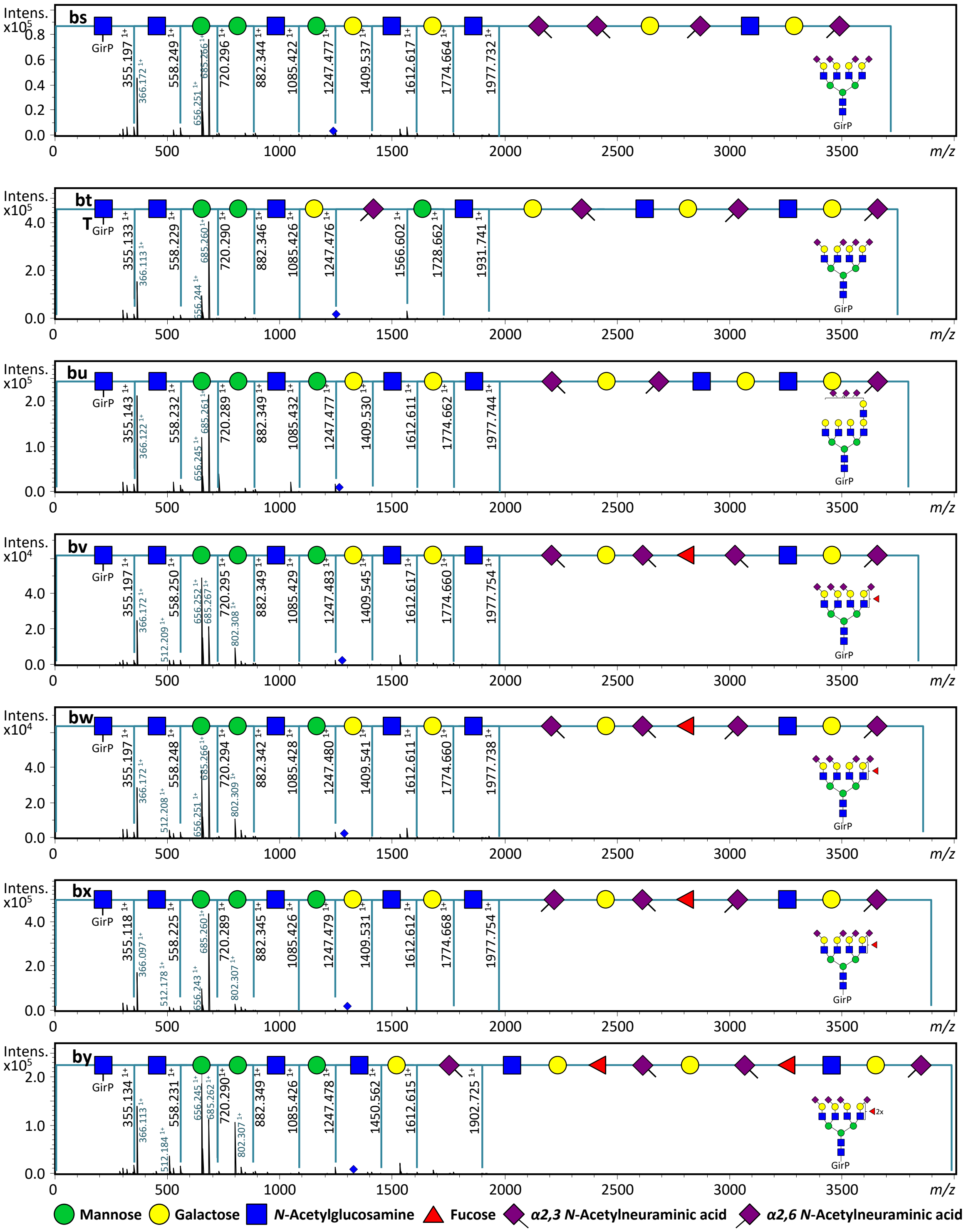

Supplementary Figure 6 (continued). CE-ESI-MS/MS fragmentation spectra of derivatized and labelled *N*-glycans. The following precursor ions were fragmented **(bs)** 1241.802 $^{3+}$ (H7N6S$_{2,3}$2S$_{2,6}$2), **(bt)** 1251.812 $^{3+}$ (H7N6S$_{2,3}$1S$_{2,6}$3) **(bu)** 1267.144 $^{3+}$ (H8N7S$_{2,3}$1S$_{2,6}$2), **(bv)** 1280.814 $^{3+}$ (H7N6F1S$_{2,3}$3S$_{2,6}$1), **(bw)** 1290.488 $^{3+}$ (H7N6F1S$_{2,3}$2S$_{2,6}$2), **(bx)** 1300.496 $^{3+}$ (H7N6F1S$_{2,3}$1S$_{2,6}$3) and **(by)** 1329.840 $^{3+}$ (H7N6F2S$_{2,3}$3S$_{2,6}$1). Blue diamond: precursor ion, blue square: *N*-acetylglucosamine, green circle: mannose, yellow circle: galactose, red triangle: fucose, right pointing pink diamond: α2,6-linked *N*-acetylneuraminic acid, left pointing pink diamond: α2,3-linked *N*-acetylneuraminic acid, GirP: Girard's reagent P label.

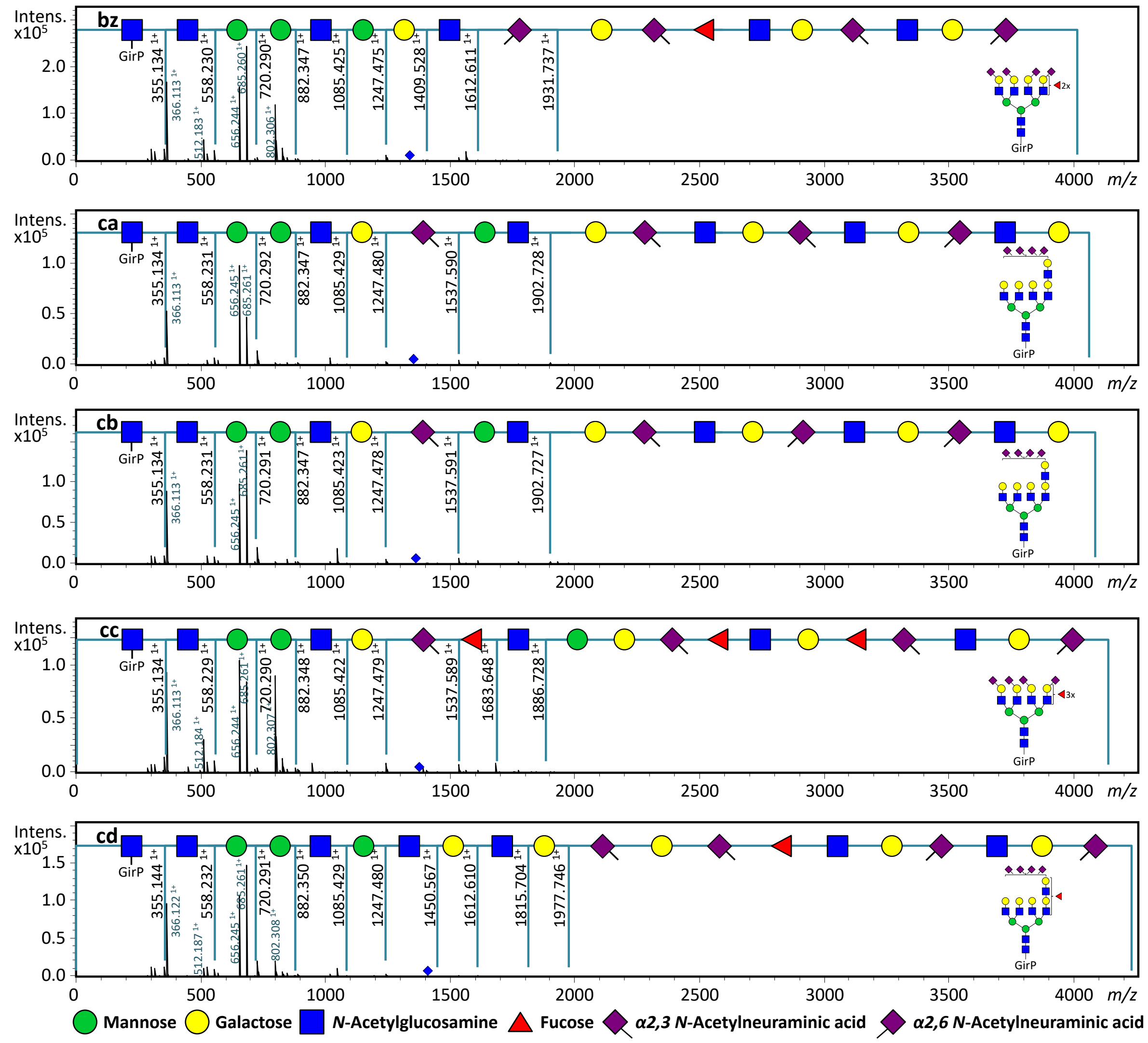

Supplementary Figure 6 (continued). CE-ESI-MS/MS fragmentation spectra of derivatized and labelled *N*-glycans. The following precursor ions were fragmented **(bz)** 1339.510 $^{3+}$ (H7N6F2S$_{2,3}$2S$_{2,6}$2), **(ca)** 1354.178 $^{3+}$ (H8N7S$_{2,3}$3S$_{2,6}$1) **(cb)** 1363.851 $^{3+}$ (H8N7S$_{2,3}$2S$_{2,6}$2), **(cc)** 1378.526 $^{3+}$ (H7N6F3S$_{2,3}$3S$_{2,6}$1), and **(cd)** 1412.533 $^{3+}$ (H8N7F1S$_{2,3}$2S$_{2,6}$2). Blue diamond: precursor ion, blue square: *N*-acetylglucosamine, green circle: mannose, yellow circle: galactose, red triangle: fucose, right pointing pink diamond: α2,6-linked *N*-acetylneuraminic acid, left pointing pink diamond: α2,3-linked *N*-acetylneuraminic acid, GirP: Girard's reagent P label.

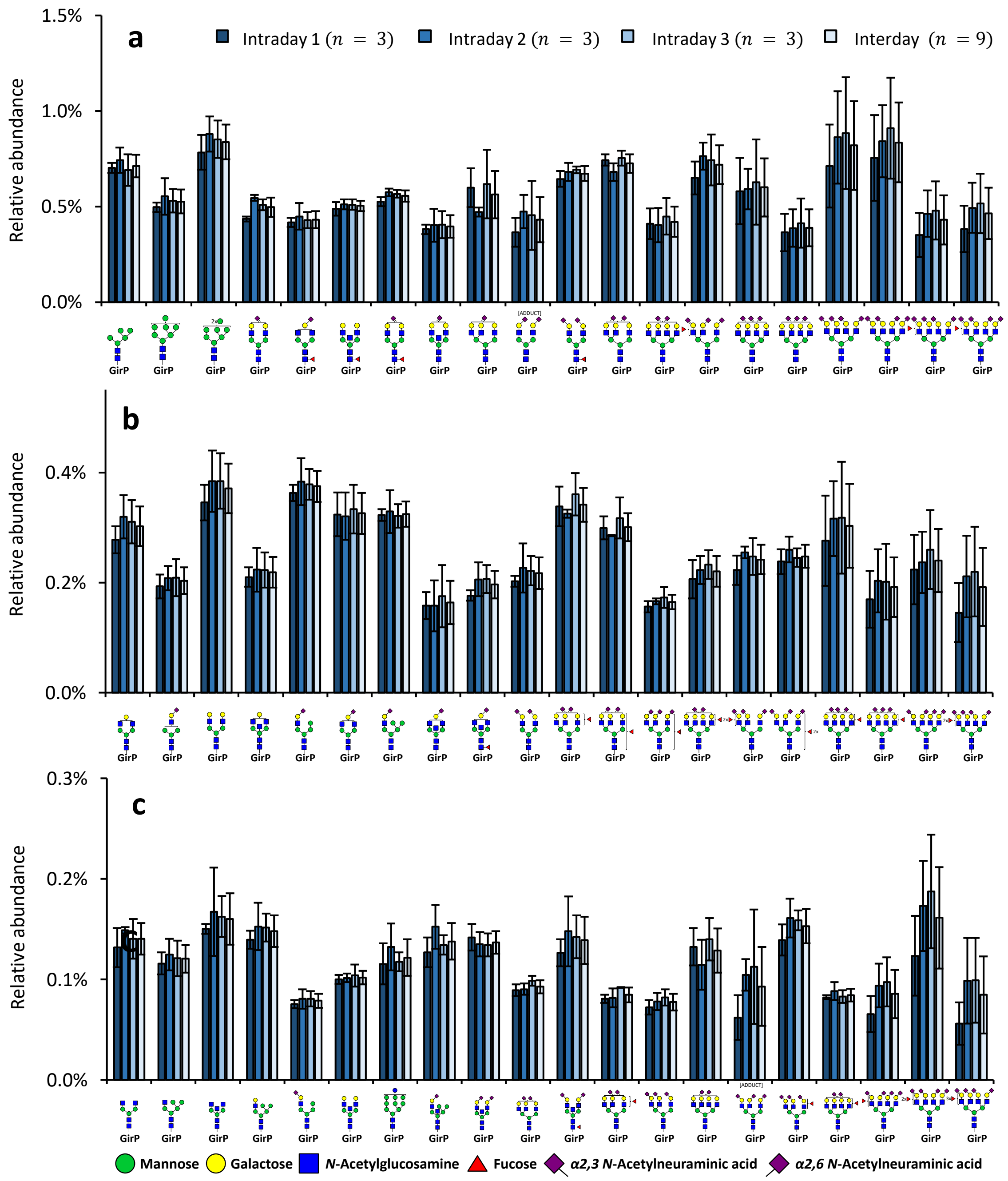

Supplementary Figure 7. Repeatability and intermediate precision of the CE-ESI-MS analysis. The released *N*-glycans were analysed after sialic acid derivatization and reducing end labelling with GirP. A total of 118 *N*-glycans could be relatively quantified using LaCyTools.[3] **(a)** Top 21-40, **(b)** 41-60 and **(c)** 61-80 *N*-glycans. Each subfigure was ordered based on the mass of the *N*-glycans (low mass to high mass). Error bars represent the standard deviation (intraday $n = 3$, interday $n = 9$, independent technical experiments). Blue square: *N*-acetylglucosamine, green circle: mannose, yellow circle: galactose, red triangle: fucose, right pointing pink diamond: α2,6-linked *N*-acetylneuraminic acid, left pointing pink diamond: α2,3-linked *N*-acetylneuraminic acid, GirP: Girard's reagent P label. Source data are provided as a Source Data file.

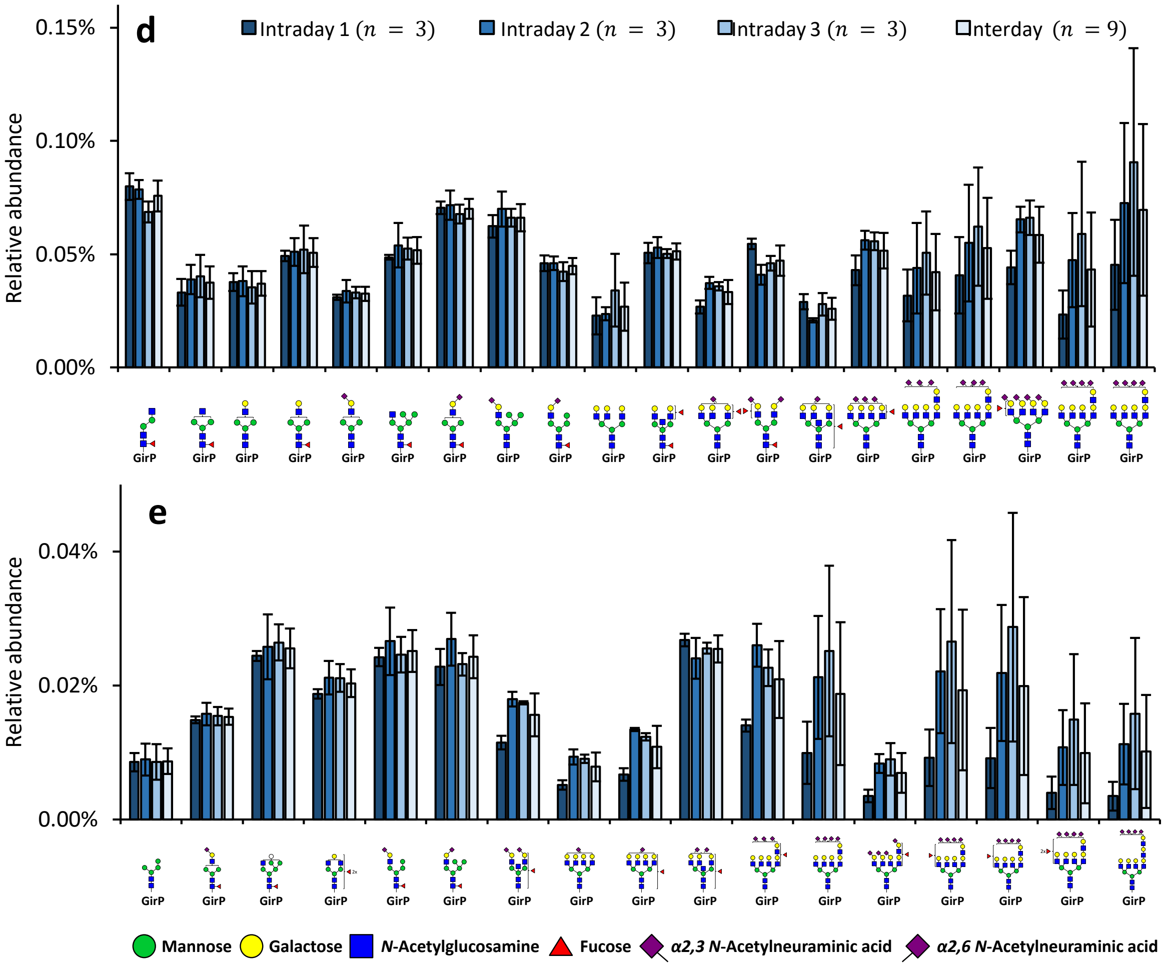

Supplementary Figure 7 (continued). Repeatability and intermediate precision of the CE-ESI-MS. The released *N*-glycans were analysed after sialic acid derivatization and reducing end labelling with GirP. A total of 118 *N*-glycans could be relatively quantified using LaCyTools.[3] **(d)** Top 81-100 and **(e)** 101-118 *N*-glycans. Each subfigure was ordered based on the mass of the *N*-glycans (low mass to high mass). Error bars represent the standard deviation (intraday $n = 3$, interday $n = 9$, independent technical experiments). Blue square: *N*-acetylglucosamine, green circle: mannose, yellow circle: galactose, red triangle: fucose, right pointing pink diamond: α2,6-linked *N*-acetylneuraminic acid, left pointing pink diamond: α2,3-linked *N*-acetylneuraminic acid, GirP: Girard's reagent P label. Source data are provided as a Source Data file.

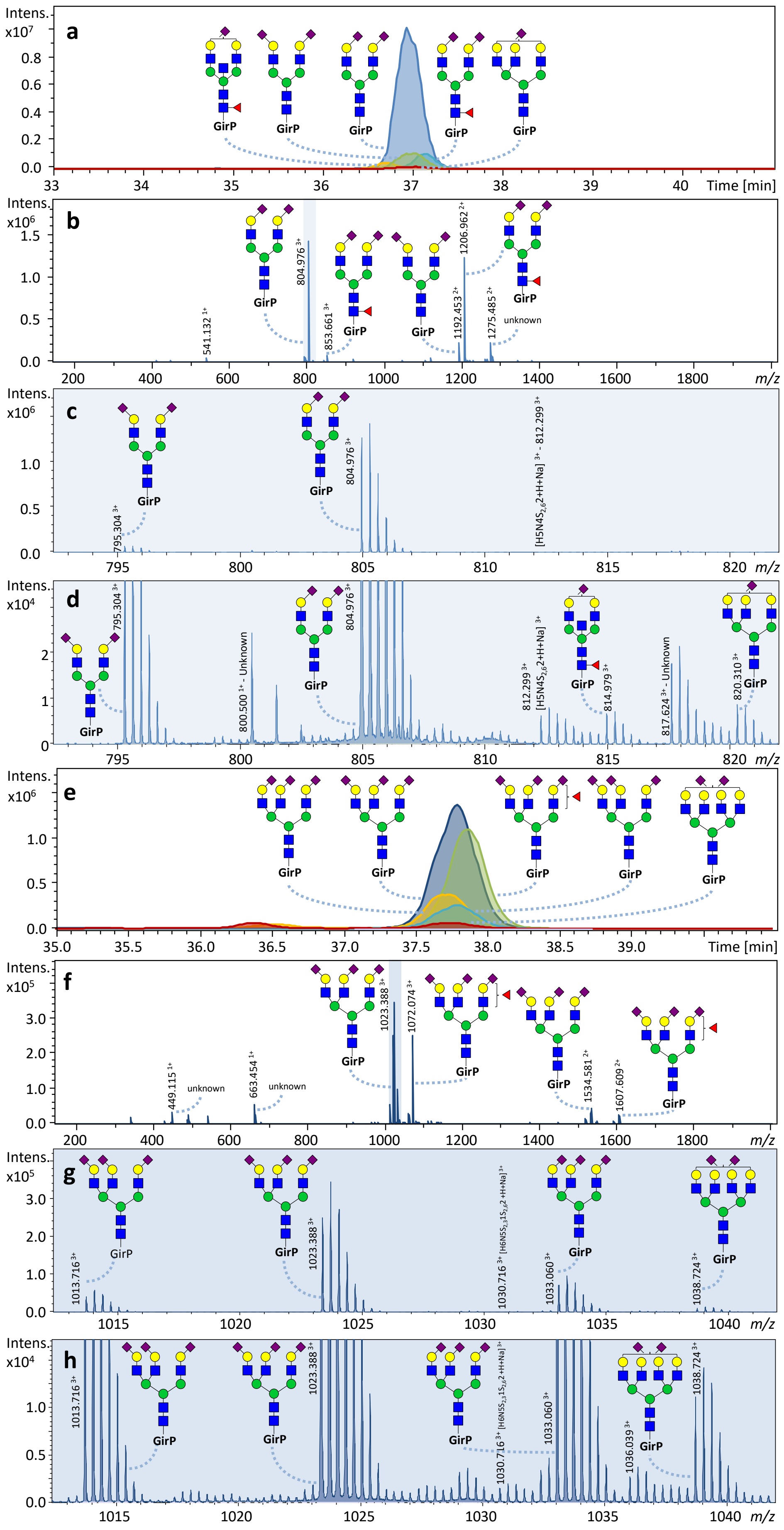

Supplementary Figure 8. Minimal (<1%) cation adduct formation is observed during MS analysis. (a and e) Extracted ion electropherograms of the highest abundant glycoform: $H5N4S_{2,6}2$, and a high abundant tri-antennary glycan: $H6N5S_{2,3}1S_{2,6}2$. **(b and f)** Summed mass spectra over migration time 36.7 – 37.2 min ($H5N4S_{2,6}2$) and 37.5 – 38.1 ($H6N5S_{2,3}1S_{2,6}2$). **(c and b)** Zoom-in on *m/z* 792 – 822 ($H5N4S_{2,6}2$) and *m/z* 1012 – 1042 ($H6N5S_{2,3}1S_{2,6}2$), indicating the detected ions underneath the extracted ion electropherogram for this m/z region. Next to the protonated species, exclusively the cation adducts $[M+H+Na]^{3+}$ were found **(d**; *m/z* 812.299 $^{3+}$ and **h**; 1030.716 $^{3+}$**)**, with a relative abundance of <1% as compared to the protonated species $[M+2H]^{3+}$. Blue square: *N*-acetylglucosamine, green circle: mannose, yellow circle: galactose, red triangle: fucose, right pointing pink diamond: α2,6-linked *N*-acetylneuraminic acid, left pointing pink diamond: α2,3-linked *N*-acetylneuraminic acid, GirP: Girard's reagent P label.

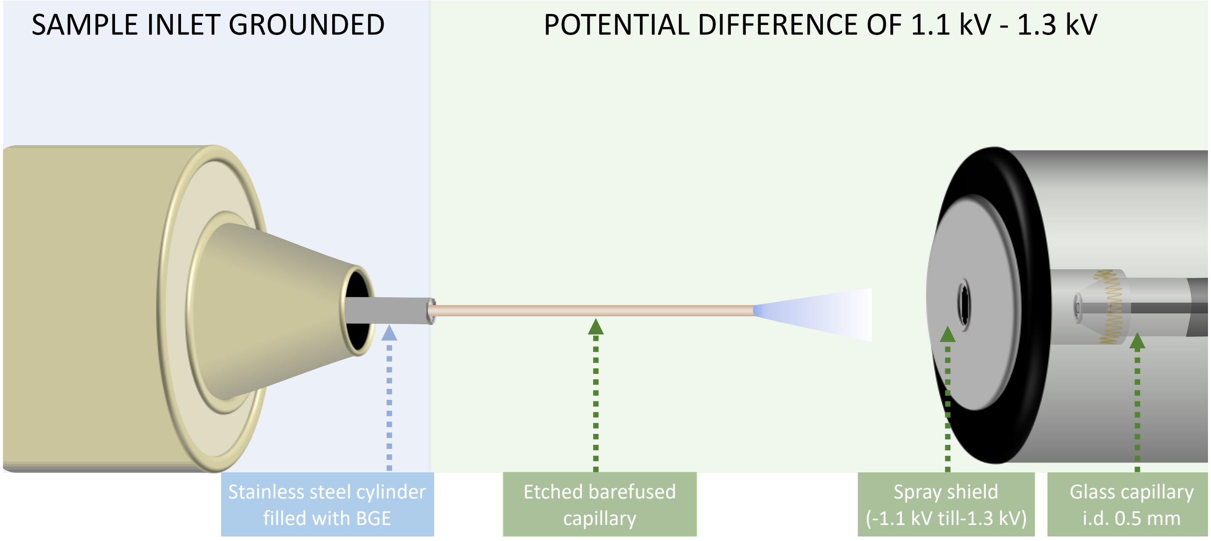

Supplementary Figure 9. Schematic overview of the interface between the CE and MS via ESI. After separation with the CE (CESI 8000, Sciex) a spray is generated at the end of the etched bare fused capillary tip. Due to the very low flow rate fine droplets are created.[4] To achieve ESI, an electrical field is created between the CE (ground potential) and the negatively charged spray shield of the MS (-1.1 kV up to -1.3 kV; Impact HD UHR-QqTOF-MS, Bruker Daltonics). By the generated electrical field the ions are attracted towards the inlet of the MS (negatively biased metal-coated glass capillary). To assist the desolvation process, nitrogen gas is heated up to 100 °C at a flow rate of 1.2 L min^{-1} which surrounds the glass capillary and the back of the spray shield.

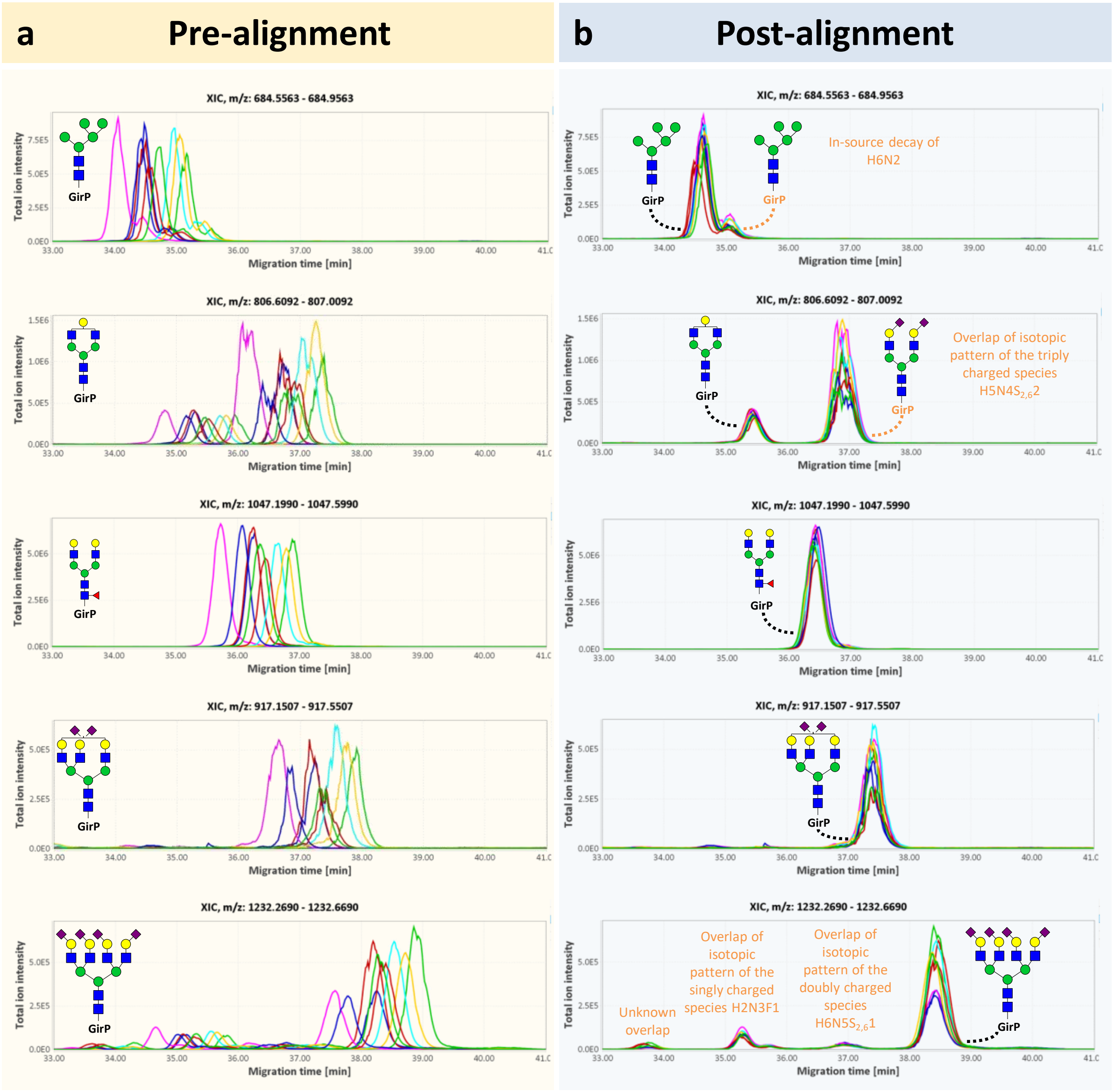

Supplementary Figure 10. Representative EIEs of glycoforms before and after alignment. (a) Raw data prior to alignment. **(b)** Aligned extracted ion electropherograms. Electropherograms can be aligned in a straightforward manner using the open source software LacyTools.[3] Although alignment is essential for automated data integration, the alignment did not show to have an effect on the robustness of the relative glycan profiles obtained (see **Supplementary Figure 11**). Visualization of the extracted ion electropherograms are made via MZmine (v2.30). Blue square: *N*-acetylglucosamine, green circle: mannose, yellow circle: galactose, red triangle: fucose, right pointing pink diamond: α2,6-linked *N*-acetylneuraminic acid, left pointing pink diamond: α2,3-linked *N*-acetylneuraminic acid, GirP: Girard's reagent P label. Source data are provided as a Source Data file.

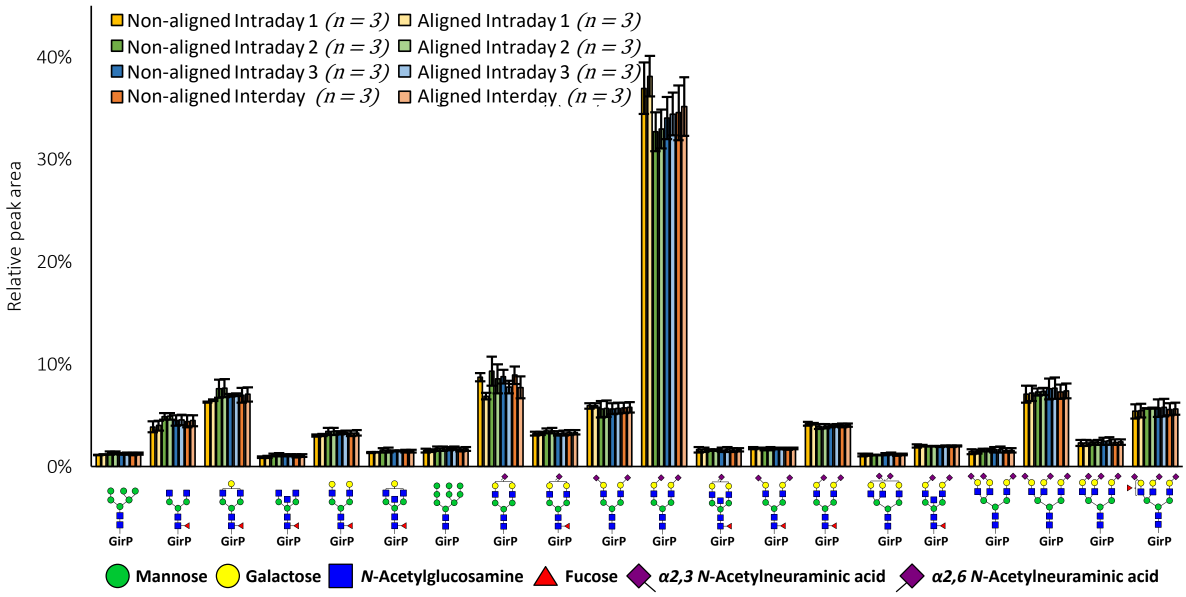

Supplementary Figure 11. Inter- and intraday repeatability before and after alignment. . The relative abundances of the 20 most abundant glycoforms are displayed, showing a median RSD of 6.9% and 4.9% within technical replicates from the same day ($n = 3$, independent technical experiments) before and after alignment, respectively. The median RSD over three days ($n = 9$, independent technical experiments) was found to be 9.7% and 9.5%, before and after alignment, respectively. The relative glycan profiles and their variation were similar before (dark coloured bars) and after (light coloured bars) alignment. Blue square: N-acetylglucosamine, green circle: mannose, yellow circle: galactose, red triangle: fucose, right pointing pink diamond: α2,6-linked *N*-acetylneuraminic acid, left pointing pink diamond: α2,3-linked *N*-acetylneuraminic acid, GirP: Girard's reagent P label. Error bars represent the standard deviation. Source data are provided as a Source Data file.

SUPPLEMENTARY TABLES

Supplementary Table 1. Optimization of the Girard's reagent P reducing end labelling of released *N*-glycans. Source data are provided as a Source Data file.

			Experimental conditions						Results	
**Variables optimized** [A]	**Sialic acid derivatization** [B]	**Number of samples**	**Original glycan sample volume (µL)**	**Reagent volume (µL)**	**Fraction EtOH in reagent**	**Fraction water in reagent**	**Concentration Girard's reagent P (mM)**	**Incubation time (h)**	**Labelling efficiency (average ± SD)** [C]	**Condition used for further optimization**
**EtOH fraction in reagent**	DA	3	5	95	65%	25%	15	2	22% ± 7%	no
	DA	3	5	95	75%	15%	5	2	24% ± 7%	no
	DA	3	5	95	85%	5%	15	2	49% ± 7%	no
	DA	3	5	95	90%	0%	15	2	52% ± 7%	yes
**Girard's reagent P concentration**	DA	3	5	95	90%	0%	15	2	58% ± 5%	no
	DA	2	5	95	90%	0%	22	2	63% ± 4%	no
	DA	3	5	95	90%	0%	33	2	68% ± 5%	no
	DA	3	5	95	90%	0%	50	2	72% ± 4%	yes
**Reagent volume and glycan sample volume**	EEA	3	5	95	90%	0%	50	2	73% ± 5%	no
	EEA	3	5	50	90%	0%	50	2	66% ± 5%	no
	EEA	3	5	20	90%	0%	50	2	36% ± 4%	no
	EEA	3	dried (5 originally)	95	90%	0%	50	2	81% ± 5%	no
	EEA	3	dried (5 originally)	50	90%	0%	50	2	83% ± 5%	no
	EEA	3	dried (5 originally)	20	90%	0%	50	2	85% ± 4%	yes
**Reagent volume**	EEA	3	dried (5 originally)	20	90%	0%	50	2	92% ± 3%	no
	EEA	3	dried (5 originally)	10	90%	0%	50	2	91% ± 4%	no
	EEA	3	dried (5 originally)	5	90%	0%	50	2	87% ± 5%	no
	EEA	3	dried (5 originally)	2	90%	0%	50	2	89% ± 5%	yes
**Incubation time**	EEA	6	dried (5 originally)	2	90%	0%	50	2	85% ± 5%	no
	EEA	6	dried (5 originally)	2	90%	0%	50	1	87% ± 4%	Final conditions [D]

A The Girard's reagent P reagent contained 10% HAc in all cases. All samples were incubated at 60 ˚C.

B Double-amidation (DA)[2] or ethyl esterification and amidation (EEA; this manuscript) of the sialic acid resulted in comparable starting products for Girard's reagent P labelling of the reducing end.

C Labelling efficiency was calculated by evaluating seven glycoforms (H4N4F1, H5N4F1, H5N4S$_{2,6}$1, H5N4F1S$_{2,6}$1, H5N4S$_{2,3}$1S$_{2,6}$1, H5N4S$_{2,6}$2, H6N5S$_{2,3}$1S$_{2,6}$2) that were reliable quantified in both their labeled and their non-labeled form in all spectra (**Supplementary Figure 3**). Averages and standard deviations of the average labelling of the seven glycoforms in the replicates are shown.

D The final conditions were used for the preparation of all samples measured by CE-ESI-MS.

Supplementary Table 2. Sensitivity assessment ($n = 3, independent\ experiments$) of the GirP labelled *N*-glycan standards with CE-ESI-MS. The light blue color highlights the values corresponding to the lowest amounts of material used that could be reliably quantified. The starting volume for all samples was 3 µL. Source data are provided as a Source Data file.

Dilution factor	Amount in reaction	Concentration after GirP labeling (in 10 µL)	Concentration after dilution with LE (in 12 µL)	Amount consumed for CE-ESI-MS measurement (in 43 nL)
1	10000 fmol	1000 fmol/µL	900 fmol/µL	39.1 fmol
10	1000 fmol	100 fmol/µL	90.0 fmol/µL	3.91 fmol
20	500 fmol	50 fmol/µL	45.0 fmol/µL	1.96 fmol
100	100 fmol	10 fmol/µL	9.00 fmol/µL	0.39 fmol
200	50 fmol	5 fmol/µL	4.50 fmol/µL	0.20 fmol
1000	10 fmol	1 fmol/µL	0.90 fmol/µL	39.13 amol
2000	5 fmol	0.5 fmol/µL	0.45 fmol/µL	19.57 amol
10000	1 fmol	0.1 fmol/µL	0.09 fmol/µL	3.91 amol

Supplementary Table 3. Overview of the diagnostic B- and Y-ions obtained by MS/MS analysis and their proposed compositions. Blue square: *N*-acetylglucosamine, green circle: mannose, yellow circle: galactose, red triangle: fucose, right pointing pink diamond: α2,6-linked *N*-acetylneuraminic acid, left pointing pink diamond: α2,3-linked *N*-acetylneuraminic acid, GirP: Girard's reagent P label.

	**Y-ions**								
**Proposed composition**	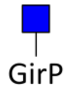	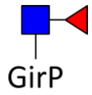	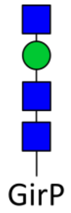	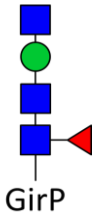	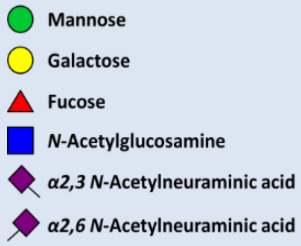				
**observed m/z**	355.162 $^{1+}$	501.220 $^{1+}$	923.373 $^{1+}$	1069.431 $^{1+}$					
	**Oxonium ions (B-ions)**								
**Proposed composition**	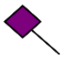	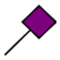	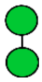	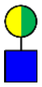	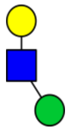	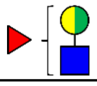	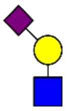	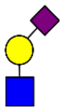	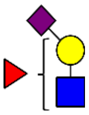
**observed m/z**	291.119 $^{1+}$	320.134 $^{1+}$	325.113 $^{1+}$	366.140 $^{1+}$	528.179 $^{1+}$	512.197 $^{1+}$	656.251 $^{1+}$	685.260 $^{1+}$	802.309 $^{1+}$

Supplementary Table 4. High-sensitivity detection of the five lowest abundant quantified glycoforms by CE-ESI-MS with DEN-gas. The most abundant glycoform and a medium abundant glycoform are also illustrated. Estimation of the overall amount of *N*-glycans in human plasma are based on the concentrations of the 24 most abundant plasma glycoproteins and the knowledge about their *N*-linked glycosylation sites as reported by Clerc *et al.*[5] This resulted in an estimation of an overall concentration of 0.64 mM *N*-glycans in human plasma. * For the CE-ESI-MS analysis 1.5 µL of the prepared sample was used with the addition of 8.5 µL leading electrolyte (LE). The light blue color highlights the values corresponding to the lowest abundant glycoform that could be reliably quantified. N: *N*-acetylhexosamine, H: hexose, F: fucose, $S_{2,6}$: α2,6-linked *N*-acetylneuraminic acid, $S_{2,3}$: α2,3-linked *N*-acetylneuraminic acid. Source data are provided as a Source Data file.

Glycan	Relative abundance	Amount in plasma (0.2 µL)	Concentration after GirP labeling (in 10 µL)	Concentration after dilution with LE *	Amount consumed for CE-ESI-MS measurement (in 43 nL)
H8N7F1$S_{2,3}$4	0.007%	8.95 fmol	0.89 fmol/µL	0.13 fmol/µL	5.84 amol
H7N6$S_{2,3}$1	0.008%	10.23 fmol	1.02 fmol/µL	0.15 fmol/µL	6.67 amol
H4N2	0.009%	11.51 fmol	1.15 fmol/µL	0.17 fmol/µL	7.50 amol
H9N8$S_{2,3}$2$S_{2,6}$2	0.010%	12.78 fmol	1.28 fmol/µL	0.19 fmol/µL	8.34 amol
H8N7F2$S_{2,3}$2$S_{2,6}$2	0.010%	12.78 fmol	1.28 fmol/µL	0.19 fmol/µL	8.34 amol
H6N5F2$S_{2,3}$1$S_{2,6}$2	5.87%	7.50 pmol	0.75 pmol/µL	0.11 pmol/µL	4.89 fmol
H5N4$S_{2,6}$2	28.05%	35.86 pmol	3.59 pmol/µL	0.54 pmol/µL	23.39 fmol

SUPPLEMENTRARY REFERENCES

(1) Reiding, K.R., et al. High-throughput profiling of protein N-glycosylation by MALDI-TOF-MS employing linkage-specific sialic acid esterification, Anal. Chem., 86, 5784-5793, **(2014)**.

(2) Holst, S., et al. Linkage-Specific in Situ Sialic Acid Derivatization for N-Glycan Mass Spectrometry Imaging of Formalin-Fixed Paraffin-Embedded Tissues, Anal. Chem., 88, 5904-5913, **(2016)**.

(3) Jansen, B.C., et al. LaCyTools: A Targeted Liquid Chromatography-Mass Spectrometry Data Processing Package for Relative Quantitation of Glycopeptides, J. Proteome Res., 15, 2198-2210, **(2016)**.

(4) Busnel, J.M., et al. High capacity capillary electrophoresis-electrospray ionization mass spectrometry: coupling a porous sheathless interface with transient-isotachophoresis, Anal. Chem., 82, 9476-9483, **(2010)**.

(5) Clerc, F., et al. Human plasma protein N-glycosylation, Glycoconj. J., 33, 309-343, **(2016)**.
