# Supplementary material for: Highly sensitive CE-ESI-MS analysis of N-glycans from complex biological samples
Source: Nat Commun. 2019 May 13;10:2137. doi: 10.1038/s41467-019-09910-7 (PMC6513864; doi:10.1038/s41467-019-09910-7)
Supplement: Supplementary file 3 — Description of Additional Supplementary Files [file 41467_2019_9910_MOESM3_ESM.docx]

**Description of Additional Supplementary Files**

**File Name: Supplementary Data 1**

**Description:** Overview of the 167 TPNG glycoforms detected by the CE-ESI-MS platform. In total, 82 glycan compositions were confirmed by fragmentation, for the remaining 85 glycan compositions confirmation was based on accurate mass (< ± 10 ppm), isotopic pattern (< 20% deviation) and migration behaviour. For the graphical representation of the *N*-glycan compositions, except from the sialic acid linkage-isomers, the monosaccharide linkages were not determined. Proposed glycan structures are based on fragmentation and literature. In addition to the CE-ESI-MS analysis, the TPNG sample was explored by a MALDI-TOF-MS platform, detecting 76 glycan compositions, based on accurate mass (< ± 20 ppm) and isotopic pattern (< 20% deviation). Finally, we compared our findings to recent literature on TPNG and IgG *N*-glycomics. Blue square: *N*-acetylglucosamine, green circle: mannose, yellow circle: galactose, red triangle: fucose, right pointing pink diamond: α2,6-linked *N*-acetylneuraminic acid, left pointing pink diamond: α2,3-linked *N*-acetylneuraminic acid, GirP: Girard’s reagent P label. N: *N*-acetylhexosamine, H: hexose, F: fucose, S2,6: α2,6-linked *N*-acetylneuraminic acid, S2,3: α2,3-linked *N*-acetylneuraminic acid.

**File Name: Supplementary Data 2**

**Description:** Overview of the TPNG and IgG *N*-glycans reported in literature, but not observed in this study. N: *N*-acetylhexosamine, H: hexose, F: fucose, S2,6: α2,6-linked *N*-acetylneuraminic acid, S2,3: α2,3-linked *N*-acetylneuraminic acid.
